# Integrated mapping and characterization of the gene underlying the okra leaf trait in *Gossypium hirsutum* L

**DOI:** 10.1093/jxb/erv494

**Published:** 2015-11-12

**Authors:** Qian-Hao Zhu, Jian Zhang, Dexin Liu, Warwick Stiller, Dajun Liu, Zhengsheng Zhang, Danny Llewellyn, Iain Wilson

**Affiliations:** ^1^CSIRO Agriculture, Black Mountain Laboratories, ACT 2601, Australia; ^2^College of Agronomy and Biotechnology, Southwest University, Chongqing 400716, PR China; ^3^CSIRO Agriculture, Locked Bag 59, Narrabri NSW 2390, Australia

**Keywords:** Cotton (*Gossypium* spp.), fine mapping, homeodomain leucine-zipper class I transcription factor, non-reciprocal homoeologous recombination, okra leaf morphology, targeted association analysis.

## Abstract

Diverse leaf morphology has been observed among accessions of *Gossypium hirsutum*, including okra leaf, which has advantages and disadvantages in cotton production. The okra leaf locus has been mapped to chromosome 15 of the D_t_ subgenome, but the underlying gene has yet to be identified. In this study, we used a combination of targeted association analysis, F_2_ population-based fine mapping, and comparative sequencing of orthologues to identify a candidate gene underlying the okra leaf trait in *G. hirsutum*. The okra leaf gene identified, *GhOKRA*, encoded a homeodomain leucine-zipper class I protein, whose closely related genes in several other plant species have been shown to be involved in regulating leaf morphology. The transcript levels of *GhOKRA* in shoot apices were positively correlated with the phenotypic expression of the okra leaf trait. Of the multiple sequence variations observed in the coding region among *GrOKRA* of *Gossypium raimondii* and *GhOKRA-D*_*t*_ of normal and okra/superokra leaf *G. hirsutum* accessions, a non-synonymous substitution near the N terminus and the variable protein sequences at the C terminus may be related to the leaf shape difference. Our results suggest that both transcription and protein activity of *GhOKRA* may be involved in regulating leaf shape. Furthermore, we found that non-reciprocal homoeologous recombination, or gene conversion, may have played a role in the origin of the okra leaf allele. Our results provided tools for further investigating and understanding the fundamental biological processes that are responsible for the cotton leaf shape variation and will help in the design of cotton plants with an ideal leaf shape for enhanced cotton production.

## Highlight

Characterization of *GhOKRA* suggests the involvement of protein activity and transcription of *GhOKRA* in regulating cotton leaf shape and a possible origin of the okra leaf trait by gene conversion.

## Introduction

Leaves are the main photosynthetic organs of vascular plants. Leaf morphology can significantly affect canopy penetration of both sunlight and chemicals applied to control insect pests, evapotranspiration, and pest preference, and consequently yield and quality of crops. Cotton is the most important nature textile fibre crop in the world. The cotton genus (*Gossypium*) consists of 50 species, comprising 45 diploids and five allotetraploids. Two A-genome diploid species [*Gossypium arboreum* (A_2_) and *Gossypium herbaceum* (A_1_)] and two AD-genome tetraploid species [*Gossypium hirsutum* (AD_1_) and *Gossypium barbadense* (AD_2_)] were independently domesticated and are cultivated for their fibres (Wendel, 1989). Cultivated cottons are dominated by *G. hirsutum* and to a much lesser extent *G. barbadense*, which are thought to have originated from relatively recent interspecific hybridization events between an A-genome-like ancestral species similar to modern *G. arboreum* or *G. herbaceum* and a D-genome-like species similar to modern *Gossypium raimondii* (D_5_; Wendel and Cronn, 2003). The leaf shape of most of the *G. hirsutum*, or upland cotton, varieties is designated as normal or palmate with from three to five rather shallow sinuses, but other leaf shapes such as subokra, okra and superokra with variable depth of indentations also exist. The leaf shape of *G. barbadense* is defined as Sea Island-type, and is similar to subokra observed in *G. hirsutum*, having moderate indentations.

Okra leaf has been associated with commercial production advantages such as accelerated flowering rates and early maturity, reduced incidence of boll rot and lint trash, increased resistance to whitefly and pink bollworm, and higher efficacy of foliar chemical application (Andries *et al.*, 1969; Thomson *et al.*, 1987; Naranjo and Martin, 1993; Heitholt and Meredith, 1998). However, reduced leaf area leads to suboptimal light capture and reduced photosynthetic rates, causing higher rates of boll shedding and often a lower yield potential under optimal conditions (Wells and Meredith, 1986; Wilson, 1986). Although a number of studies have been performed, it is still unclear whether the yield deficiency observed in the okra leaf varieties is contributed by the gene underlying the okra leaf trait or due to disadvantageous linkage drag associated with the okra leaf locus, although some Australian okra leaf cultivars are as productive as normal leaf varieties (Thomson, 1994). Identifying the gene determining the okra leaf trait and a better understanding of the regulatory networks associated with leaf morphology in cotton may provide novel tools for development of superior cotton genotypes with ideal leaf morphology and improved productivity.

To establish the genetic architecture of cotton leaf shape, cotton researchers started genetic analyses of leaf shape about a century ago (Shoemaker, 1909; Peebles and Kearney, 1928). These early studies suggested that the okra leaf trait in *G. hirsutum* is controlled by a single semi-dominant gene, as leaves of F_1_ plants derived from a cross between okra leaf and normal leaf varieties showed intermediate leaf shape, i.e. subokra. During the last two decades, many mapping populations derived from crosses of intraspecific varieties (*G. hirsutum*) or interspecific varieties (*G. hirsutum* and *G. barbadense*) have been developed and used to identify quantitative trait loci (QTLs) associated with various attributes of cotton leaf shape (Jiang *et al.*, 2000; Lacape *et al.*, 2013; Andres *et al.*, 2014; Zhu *et al.*, 2014). A large number of QTLs affecting leaf size and shape were identified on several chromosomes (Jiang *et al.*, 2000) but the major locus determining the okra leaf trait was consistently identified on chromosome 15 (Chr15) of *G. hirsutum* (Jiang *et al.*, 2000; Lacape *et al.*, 2013; Andres *et al.*, 2014; Zhu *et al.*, 2014). Recently, the okra leaf locus has been narrowed down to a 5.4 cM region in *G. hirsutum* (Andres *et al.* 2014), corresponding to a 337kb region on Chr02 (equivalent to Chr15 of *G. hirsutum*) of the *G. raimondii* (D_5_) genome (Paterson *et al.*, 2012). This region contains 34 annotated genes including two homeodomain leucine-zipper (HD-Zip) class I transcription factors, *Gorai.002G244000* and *Gorai.002G244200*, which were suggested to be the possible candidate genes determining the okra leaf shape (Andres *et al.*, 2014).

In this study, we aimed to identify the gene underlying the okra leaf shape in *G. hirsutum*. Upon confirmation of the chromosomal region containing the okra leaf locus using the CottonSNP63K array (Hulse-Kemp *et al.*, 2015), we were able to locate the okra leaf locus to a region corresponding to Chr02 of *G. raimondii* containing a single gene (*Gorai.002G244000*) using a combination of targeted association analysis and traditional F_2_ population-based genetic mapping. Based on the function of the genes closely related to *Gorai.002G244000* in other plant species and sequence variations among *G. hirsutum* accessions showing a normal, okra, or superokra leaf phenotype, we deduced that the *G. hirsutum* orthologue of *Gorai.002G244000*, designated *GhOKRA*, is the best candidate gene determining the okra leaf trait in upland cotton. We also found that non-reciprocal homoeologous recombination (NRHR) could have played a role in the origin of the okra leaf trait.

## Materials and methods

### Plant materials

The plant materials used in this study comprised: 177 accessions of *G. hirsutum* (AD_1_) ( Supplementary Table S1 at *JXB* online); two F_2_ populations segregating for the okra leaf trait, the first containing 1873 (448 okra:964 subokra:461 normal) individuals derived from RIL034 [okra leaf, derived from T586×Yumian1 (Zhang *et al.*, 2009)]×Yumian1 (normal leaf), and the second containing 310 (72 okra:142 subokra:96 normal) individuals derived from RIL090 (okra leaf, derived from T586×Yumian1)×Jinnong08 (normal leaf); three accessions of *G. barbadense* (AD_2_); three accessions of *G. arboreum* (A_2_); and a *G. arboreum* F_2_ population (68 plants) derived from Yunnanzhongmian (YZ)×BM13H. All plants were grown in a glasshouse (Canberra, Australia) at 28±2 °C with approximately 16/8h day/night regime except for the two F_2_ populations, which were grown in the field at the Experimental Station of Southwest University (Chongqing, China). Of the 177 *G. hirsutum* accessions, 85 were used in single-nucleotide polymorphism (SNP) genotyping with the CottonSNP63K array (Hulse-Kemp *et al.*, 2015) to confirm the location of the okra leaf QTL identified in our previous study (Zhu *et al.*, 2014), and 92 were used in association analysis-based fine mapping of the okra leaf locus using KASP (Kompetitive Allele Specific PCR) assays (15 okra leaf accessions from these 92 samples were also genotyped using the CottonSNP63K array). The F_2_ population derived from YZ×BM13H was used in co-segregation analysis. Leaf shape was recorded for all materials at the six-leaf stage and then confirmed at the flowering stage.

### Preparation of DNA and RNA samples

For SNP genotyping (SNP chip and KASP assay), cotyledons were sampled and used in DNA extraction using a DNeasy Plant Mini kit (Qiagen) according to the manufacturer’s instructions. All DNA samples were quantified using NanoDrop 1000 (Thermo Scientific) and diluted to 50ng µl^–1^. For simple sequence repeat (SSR) genotyping, DNA was extracted from a young leaf of each F_2_ individual using the CTAB approach (Zhang *et al.*, 2005). RNA samples used in quantitative real-time PCR (qRT-PCR) were collected from MCU-5 (normal leaf) and Siokra 1-4 (okra leaf) at various developmental stages ( Supplementary Fig. S1 at *JXB* online) and extracted as described below. Samples were root, cotyledon, the first, third, and fifth fully expanded leaf, shoot apical meristem (SAM; including ≤1cm young developing leaves) at the first-, third-, and fifth-leaf stages, leaf margin (~0.5cm width), and the interior part (~1cm flanking the midvein) of the eighth fully expanded leaf. Total RNA was isolated using an RNeasy Plant Mini kit (Qiagen) and quantified using a Qubit-iT RNA Assay kit (Life Technologies).

### SNP and SSR genotyping

For genotyping using the SNP chip, standardized DNA at 50ng µl^–1^ for each of the *G. hirsutum* cotton accessions described above was processed according to Illumina protocols and hybridized to the CottonSNP63K array at CSIRO Agriculture (Brisbane, Australia) according to the manufacturer’s instructions. Single-base extension was performed and the chips were scanned using the Illumina iScan. Image files were saved and analysed using the GenomeStudio Genotyping Module (v.1.9.4, Illumina). Genotype calls for each SNP were based on the cluster file generated specifically for the CottonSNP63K array (Hulse-Kemp *et al.*, 2015).

For KASP genotyping, the DNA concentration of each sample was standardized to 15ng µl^–1^. The amplification was performed in an 8 µl reaction, comprising 1 µl of DNA, 4 µl of 2× KASP master mix (LGC Group), 0.11 µl of primer mix (12 µM of each allele-specific primer and 30 µM of common primer) and 2.89 µl of H_2_O, according to the manufacturer’s instructions. Assays were performed in 384-well format. PCR cycling was performed on an Eppendof Mastercycler ep384 using the following protocol: hotstart at 95 °C for 15min, followed by 10 touchdown cycles (95 °C for 20s; touchdown at 65–57 °C, 0.8 °C per cycle, 60s), and then followed by 31 cycles of amplification (94 °C for 20s; 57 °C for 60s). Plates were read on the ViiA7 Real-Time PCR System (Life Technologies) at ambient temperature and analysed using the Applied Biosystems software. If discriminating genotyping clusters had not formed after the initial amplification, an additional three to six cycles of amplification were conducted and the plate was read and analysed again. Primers used in the KASP analyses are shown in Supplementary Table S2 at *JXB* online.

**Fig. 1.**
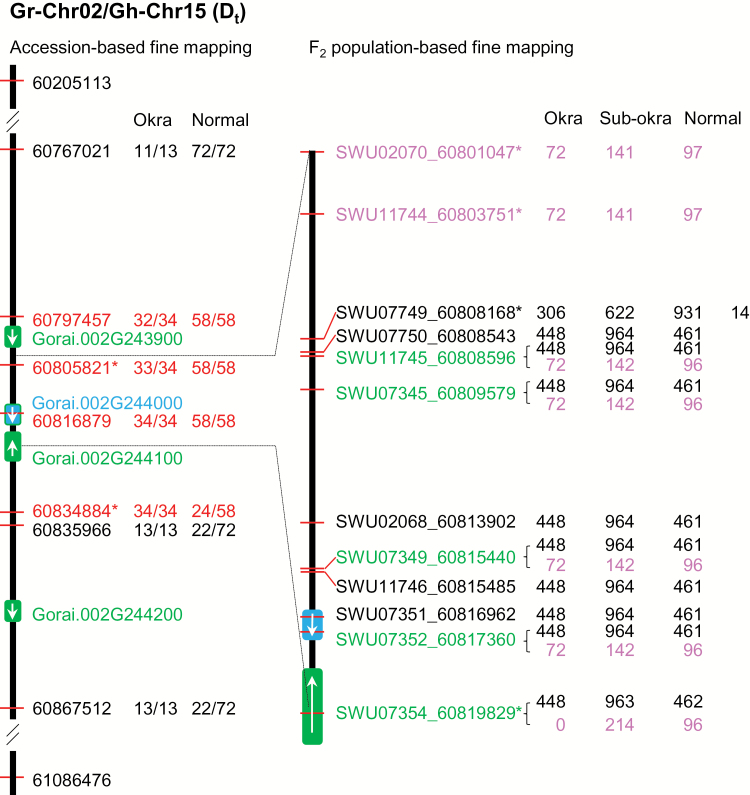
Fine mapping of the okra leaf locus in *G. hirsutum*. The okra leaf locus was mapped previously on Chr15 of *G. hirsutum* corresponding to the region between 60 205 113 and 61 086 476 on Chr02 of *G. raimondii* using an F_7_ recombinant inbred line population (Zhu *et al.*, 2014). In this study, two strategies that used *G. hirsutum* accessions or F_2_ populations were used to fine map the okra leaf locus. In the accession-based approach, the okra leaf locus was first narrowed down to the region (~69kb) between 60 767 021 and 60 835 966 using 85 *G. hirsutum* accessions showing normal (*n*=72) or okra leaf (*n*=13) shape. Using SNP markers located within the ~69kb region and another set (*n*=92) of *G. hirsutum* accessions, the okra leaf locus was further mapped to the interval (between 60 805 821 and 60 834 884) with just two annotated genes, *Gorai.002G244000* and *Gorai.002G244100*. In the F_2_-based genetic linkage analysis, the okra leaf locus was narrowed down to an ~12kb region (between SWU07749 and SWU07354) using two F_2_ populations. This interval contains only a single gene, i.e. *Gorai.002G244000*. The black vertical bars represent the chromosome. Green and blue boxes represent annotated genes. White arrows indicate the transcriptional direction of the genes. The numbers in the first column next to the black bars represent the coordinates of the SNP markers used in the CottonSNP63K (in black) and KASP (in red) assays, or of the SSR markers used in genotyping of the F_2_ populations. SSR markers in black and pink were unique to the RIL034×Yumian1 and RIL090×Jinnong08 populations, respectively, while those in green were common to the two populations. For the accession-based fine mapping, the numbers before and after the forward slash represent the number of *G. hirsutum* accessions with a consistent phenotype and genotype at the corresponding SNP position and the total number of *G. hirsutum* accessions showing okra or normal leaf shape, respectively. For the F_2_-based fine mapping, the number of plants with a genotype consistent with okra, subokra, and normal leaf shape are shown. For marker SWU07749, 14 plants did not have genotyping results. The markers with an asterisk (*) are the delimited ones for the okra leaf locus.

For SSR analysis, markers were designed based on the genome sequence of the *G. raimondii* Chr02, and primers were designed using the Primer6.0 program (http://www.premierbiosoft.com/, accessed 4 November 2015). PCR was performed in a 10 μl reaction containing 20ng template DNA, 1 μl of 10× PCR buffer, 0.2 μl of 2mM dNTPs, 0.2 μl of *Taq* DNA polymerase (5U μl^–1^), and 0.2 μl each of the forward and reverse primers (10 μΜ). The amplification program was: 5min at 94 °C; 35 cycles of 30s at 94 °C, 30s at 55 °C, and 1min at 72 °C, and a final cycle of 10min at 72 °C. The PCR products were separated in 10% polyacrylamide gels and visualized by silver staining. The PCR primers of the SSR markers shown in Fig. 1 are listed in Supplementary Table S2.

### Fine mapping using the F_2_ populations

Two F_2_ populations were used in fine mapping of the okra leaf locus. For the first F_2_ population (RIL034×Yumian1), 92 plants randomly selected from the population were first screened using 66 SSR markers that were polymorphic between RIL034 and Yumian1 to identify markers (*n*=28) showing linkage with the okra leaf trait. Those 28 markers were then used to genotype the remaining 1781 F_2_ plants to identify eight markers (SWU07750, SWU11745, SWU07345, SWU02068, SWU07349, SWU11746, SWU07351, and SWU07352) co-segregating with the okra leaf trait (Supplementary Fig. S2A at *JXB* online). For the second population (RIL090×Jinnong08), 92 plants randomly selected from the population were first screened using 32 SSR markers that were polymorphic between RIL090 and Jinnong08 to identify those (15 markers) showing linkage with the okra leaf. The remaining 208 F_2_ individuals were then genotyped using these 15 SSR markers. Four markers (SWU11745, SWU07345, SWU07349, and SWU07352) were found to be co-segregating with the okra leaf trait (Supplementary Fig. S2B). Phenotypic data and genotyping results were processed using the SPSS16.0 software package. Genetic linkage analysis was done using Joinmap 4.0 (http://www.kyazma.nl/index.php/mc.JoinMap/, accessed 4 November 2015) with the following settings: LOD=4.0, recombination rate=0.4, mapping function=Kosambi.

### Sequence comparison

The genomic sequences containing the orthologue of *Gorai.002G244000* were amplified using LS_F1 and LS_R1 (Supplementary Table S2) from MCU-5, Sicot 71, Coker 315, TM-1, 89004-64 and Siokra 1-4 (*G. hirsutum*, AD_1_), 3-79, Pima A8, and Sipima 280 (*G. barbadense*, AD_2_) using a Phusion High-Fidelity PCR kit (New England BioLabs). Promoters of *GhOKRA* in MCU-5 and Siokra 1-4 were amplified using LS_F2 and LS_R2 (Supplementary Table S2). PCR products were subcloned into the pCR^®^4Blunt-TOPO vector (Invitrogen) and eight clones were sequenced for each PCR product. LS_F1 and LS_R1, which contain three and one SNP with the corresponding sequences in the *G. arboreum* genome, respectively, were designed based on the *G. raimondii* genome sequence with the aim of amplifying only the D_t_ subgenome because the okra leaf trait had previously been mapped to the D_t_ subgenome of *G. hirsutum*. The sequences of the cloned PCR products were assigned to the D_t_ or A_t_ subgenome of *G. hirsutum* based on their similarity to the corresponding sequences from the D_5_ and A_2_ (*G. arboreum*) genomes (Paterson *et al.*, 2012; Li *et al.*, 2014). As expected, for most accessions, only the D_t_ subgenome sequence was amplified; however, the A_t_ subgenome homeologue was also amplified from 3-79 and Pima A8, probably due to less divergence between the D_t_ and A_t_ subgenomes in the primer-binding sites. The genomic sequences containing the orthologue of *Gorai.002G244000* in the A_t_ subgenome of MCU-5, Siokra 1-4, Sicot 71, and Coker 315, as well as in YZ, BM13H, and M18 (*G. arboreum*), were based on whole-genome sequencing results (unpublished data). Sequences of Yumian1 and T586 were based on sequencing of bacterial artificial chromosomes generated from these two accessions (unpublished data). The gene structure of *GhOKRA* was confirmed to be the same as that of *Gorai.002G244000* by RACE using a GeneRacer^TM^ kit (Invitrogen) and RNA isolated from the shoot apices of MCU-5. Sequence alignment was performed using ClustalW2 (http://www.ebi.ac.uk/Tools/msa/clustalw2/, accessed 4 November 2015) and the phylogenetic tree was generated using MEGA6 (http://www.megasoftware.net/, accessed 4 November 2015).

### Identification of cotton orthologues of the *Arabidopsis CUC2* gene

The protein sequence of *Arabidopsis* CUC2 (At5g53950) was used to search against the annotated *G. raimondii* and *G. arboreum* transcripts as well as genome sequences of *G. raimondii* (Paterson *et al.*, 2012) and *G. arboreum* (Li *et al.*, 2014) by tblastn with a cut-off value of *E≤*10^–100^. Three potential *CUC2* orthologues were identified in both *G. raimondii* (*Gorai.002G067300*, *Gorai.007G323900*, and *Gorai.013G171300*) and *G. arboreum* (*A_06256*, *A_17275*, and *A_16773*). Their *G. hirsutum* orthologues were identified in the recently reported *G. hirsutum* genome sequence (Zhang *et al.*, 2015; Supplementary Fig. S3 at *JXB* online). For each pair of homoeologues, a single pair of primers matching both homoeologues was used in qRT-PCR.

### Gene expression analysis using qRT-PCR

qRT-PCR was performed according to our previous protocols (Zhu *et al.*, 2013) except that the reference gene was the cotton ubiquitin gene (GenBank accession no. EU604080). The primers used in qRT-PCR analyses are shown in Supplementary Table S2. All primer pairs had a similar PCR efficiency (87.9–99.6%).

## Results

### Fine mapping of the okra leaf locus

We have previously mapped the okra leaf locus to Chr15 (D_t_ subgenome) of *G. hirsutum*, which corresponds to a region of ~880kb on *G. raimondii* Chr02 (Zhu *et al.*, 2014; Fig. 1). In this study, we used two strategies, targeted association analysis and traditional genetic linkage analysis, to fine map the okra leaf locus.

**Fig. 2.**
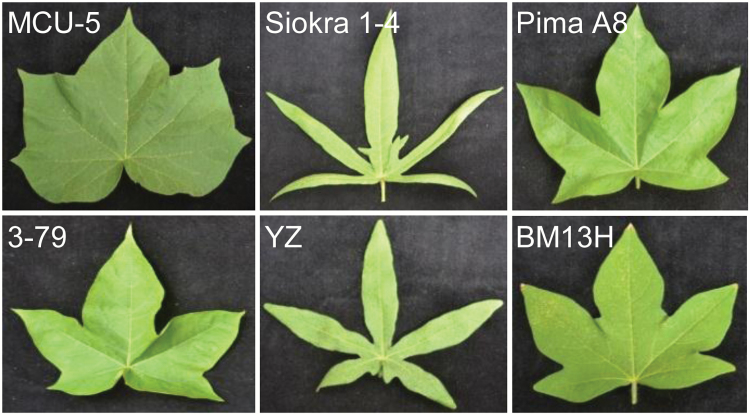
Representative mature leaves of some of the accessions used in this study. MCU-5 (normal) and Siokra 1-4 (okra) belong to *G. hirsutum* (AD_1_); Pima A8 and 3-79 (both are subokra) belong to *G. barbadense* (AD_2_); and YZ (okra) and BM13H (subokra) belong to *G. arboreum* (A_2_).

In the targeted association analysis, we first genotyped 85 *G. hirsutum* accessions showing normal (*n*=72) or okra (*n*=13) leaf using the recently developed CottonSNP63K array (Hulse-Kemp *et al.*, 2015) to confirm the region containing the okra leaf locus on Chr15. This analysis not only confirmed our previous QTL mapping result but also allowed us to narrow down the okra leaf locus to an ~69kb region (Fig. 1; between 60 767 021 and 60 835 966 of *G. raimondii* Chr02). We then identified SNPs located within the ~69kb region that were polymorphic between MCU-5 (normal leaf) and Siokra 1-4 (okra leaf) (Fig. 2) using our whole-genome sequences of these two accessions (unpublished data). KASP assays were designed for several of these SNPs and used to genotype 58 normal leaf and 34 okra leaf *G. hirsutum* accessions that were different from those used in the SNP chip assay (Supplementary Table S1). These assays allowed us to further narrow down the okra leaf locus to an ~29kb region (between 60 805 821 and 60 834 884 of *G. raimondii* Chr02), in which there are only two genes, *Gorai.002G244000* and *Gorai.002G244100* (Fig. 1, Supplementary Table S3 at *JXB* online).

In the genetic linkage analysis, we first used SSR markers designed based on the sequence of *G. raimondii* Chr02 that were polymorphic between RIL034 and Yumian1 to genotype an F_2_ population with 1873 individuals derived from RIL034×Yumian1. In this population, the okra leaf locus was mapped to an interval delimited by markers SWU07749 and SWU07354, which corresponds to an ~12kb region on *G. raimondii* Chr02. Because the north-side SSR markers, including SWU07749, showed distorted segregation ( Supplementary Fig. S2A), similar genetic linkage analysis using a second F_2_ population with 310 individuals derived from RIL090×Jinnong08 was carried out. In this population, the okra leaf locus was mapped to an interval delimited by markers SWU02070 and SWU07354, corresponding to an ~16kb region on *G. raimondii* Chr02 (Supplementary Fig. S2B). The *G. raimondii* chromosomal region identified in both F_2_ populations contains only a single gene, *Gorai.002G244000* (Fig. 1). Based on these mapping results, we reasoned that the *G. hirsutum* orthologue, designated *GhOKRA*, of *Gorai.002G244000* is the gene responsible for the okra leaf trait in upland cotton.

### Sequence diversity at the *GhOKRA* locus in different *Gossypium* accessions

**Fig. 3.**
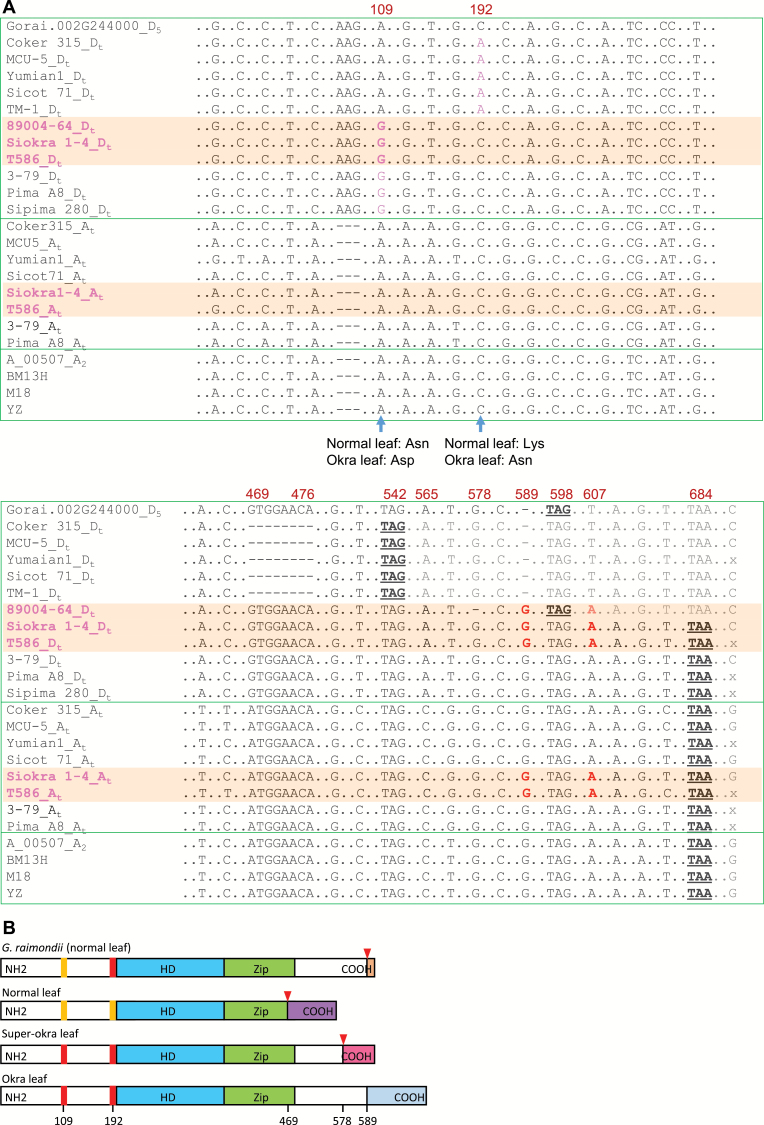
Comparison of the nucleotide and protein sequences of the okra leaf gene. (A) Alignment of the coding sequences of *Gorai.002G244000* and its orthologues in other cotton species. Only the positions that are polymorphic between any two sequences are shown. Accession name followed by D_t_ (e.g. Coker 315_D_t_) and A_t_ represents the D_t_ and A_t_ subgenome allele, respectively. Coker 315, MCU-5, Yumian1, Sicot 71, and TM-1 are normal leaf *G. hirsutum* accessions; 89004-64, Siokra 1-4, and T586 (shown in pink and highlighted) are superokra leaf or okra leaf *G. hirsutum* accessions. Pima A8, 3-79, and Sipima 280 are *G. barbadense* accessions showing the subokra leaf shape. YZ (okra), M18, and BM13H (subokra) are *G. arboreum* accessions. A dash represent a deletion. Stop codons are shown in bold and underlined. The D_t_ subgenome SNPs between the normal and okra leaf accessions and the SNPs suggesting the NRHR event are shown in pink and red, respectively. Positions shown on top of the sequences were based on *GhOKRA-D*_*t*_ of Siokra 1-4, i.e. Siokra 1-4_D_t_. An ‘x’ at the end of some sequences indicates that data were unavailable. (B) Schematic representation of GhOKRA-D_t_ from *G. raimondii* and normal, okra, and superokra leaf *G. hirsutum* accessions. Rectangles represent protein sequences with differences shown in different colours. The numbers at the bottom indicate the nucleotide locations of SNPs or indels. Red triangles indicate the positions of indel(s) that caused a frame shift of the okra leaf allele. HD, homeodomain; Zip, Zip domain.

To identify mutation(s) within *GhOKRA*, we first performed RACE to clone the cDNA of *GhOKRA* from MCU-5 (normal leaf) using RNA isolated from shoot apices. The primer used in 3′ RACE fully matched the D_5_ genome and had one mismatch in the middle of the primer with the A_2_ genome. We expected that the primer would be able to amplify both the D_t_ and A_t_ subgenome alleles; however, we were only able to amplify the D_t_ subgenome allele (*GhOKRA-D*_*t*_; Supplementary Fig. S4 at *JXB* online), suggesting that *GhOKRA-A*_*t*_ (the A_t_ subgenome allele) is not expressed or is expressed at a very low level in shoot apices. Expression levels of *GhOKRA-A*_*t*_ detected by primers specific to the A_2_ genome orthologue confirmed this assertion (data not shown). According to sequence comparisons between the cDNA and genomic sequence of *GhOKRA-D*_*t*_ from MCU-5, *GhOKRA-D*_*t*_ and *Gorai.002G244000* had the same gene structure, i.e. two introns and three exons; however, the predicted protein sequence of GhOKRA-D_t_ was 21 aa shorter than that of Gorai.002G244000 due to a frameshift mutation and premature stop codon caused by an 8bp deletion in *GhOKRA-D*_*t*_ beginning at position 469 of the *G. raimondii* sequence (Fig. 3A, Supplementary Figs S5 and S6 at *JXB* online). The genomic sequences corresponding to *GhOKRA-D*_*t*_ from another four normal leaf (Coker 315, Yumian1, Sicot 71, and TM-1), two okra leaf (Siokra 1-4 and T586), and one superokra leaf (89004-64; Supplementary Fig. S1) *G. hirsutum* accessions were sequenced, and their coding sequences were determined based on *GhOKRA-D*_*t*_ from MCU-5. All five normal leaf *G. hirsutum* accessions had identical coding sequences. The okra leaf and the superokra leaf accessions all lacked the 8bp deletion observed in the normal leaf accessions and had an insertion of a G at nt 589; therefore, their coding sequences were longer than those in the normal leaf varieties (Fig. 3A and Supplementary Fig. S5). The superokra accession, however, had a single-base deletion at nt 578 that restores the reading frame to make the predicted protein the same size as that in *G. raimondii* but with a different C terminus (Fig. 3B and Supplementary Fig. S6). More importantly, this second mutation changed the leaf shape from okra to superokra (Supplementary Fig. S1B), strongly supporting *GhOKRA-D*_*t*_ as the best candidate gene underlying leaf shape variations in *G. hirsutum*. Two non-synonymous SNPs (positions 109 and 192) were observed between the normal and okra/superokra leaf accessions, but there was no difference at position 192 between *G. raimondii* and the okra/superokra leaf *G. hirsutum* accessions (Fig. 3). As *G. raimondii* is thought to be the D_t_ subgenome donor of *G. hirsutum* and normal leaf the ancestral leaf shape, we reasoned that the sequence of *GhOKRA-D*_*t*_ in the okra leaf *G. hirsutum* accessions should at least be different from that of *G. raimondii*. The non-synonymous SNP at position 192 and the 8bp deletion between the normal and okra leaf accessions are not found in the *G. raimondii* gene, so are unlikely to be linked to the okra leaf phenotype, which leaves the non-synonymous SNP at position 109, which changed an asparagine (Asn) to an aspartate (Asp), and the protein sequence differences at the C terminus caused by the indels at positions 578 and 589 (Fig. 3B) as the likely cause(s) for the okra/superokra leaf phenotype.

The orthologous sequences of *GhOKRA-D*_*t*_ were also amplified from three *G. barbadense* accessions, all of which showed a subokra leaf shape (Fig. 2). Interestingly, all three *G. barbadense* accessions had the same coding sequences as Siokra 1-4 (Fig. 3 and Supplementary Fig. S5), suggesting that the sequence changes observed between the normal and okra leaf accessions of *G. hirsutum* had a slightly less pronounced effect on leaf shape formation in *G. barbadense*, probably due to its different genetic background.

Accessions of *G. arboreum* had diverse leaf shapes, from similar to subokra leaf (such as M18; Supplementary Fig. S1C) to okra leaf (such as YZ; Fig. 2). Although *GhOKRA-A*_*t*_ was almost undetectable in *G. hirsutum*, we reasoned that the okra leaf shape in *G. arboreum* should be defined by the orthologue of *GhOKRA-A*_*t*_, i.e. *A_00507* annotated in the *G. arboreum* genome (from Shixiya1; Li *et al.*, 2014). Nevertheless, the coding sequences of *A_00507* were identical in the three *G. arboreum* accessions (YZ, BM13H, and M18; Fig. 3) each with a different leaf shape (Fig. 2 and Supplementary Fig. S1) and to Shixiya1 (subokra leaf, X.-M. Du, personal communication), although some SNPs were found in the 5′ and 3′ UTRs between YZ and BM13H. KASP genotyping using the SNP located at ~300bp downstream of the stop codon of *A_00507* indicated that it co-segregated with the okra leaf trait in an F_2_ population derived from YZ×BM13H. These results suggest that, although we could not conclusively confirm *A_00507* as the gene determining the leaf shape trait in *G. arboreum*, it is still a good candidate, and the sequence variations observed in the 5′ and 3′ non-coding regions could play a role in determining leaf shape in *G. arboreum*.

### Expression levels of *GhOKRA* and *GhCUC2* are positively correlated with development of the okra leaf trait

**Fig. 4.**
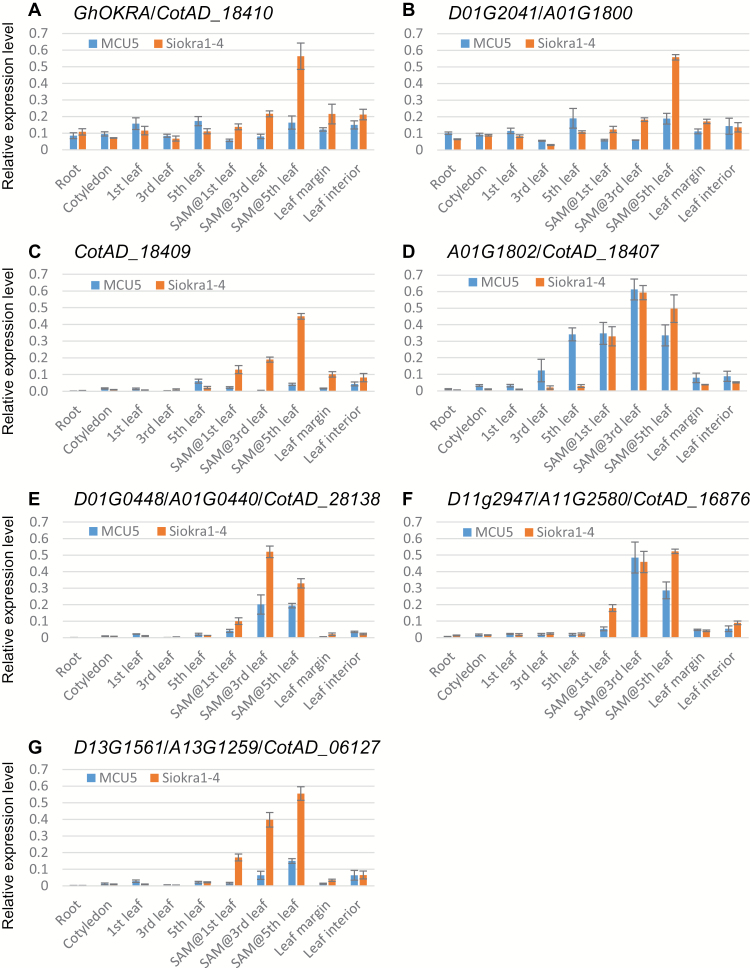
Expression levels of *GhOKRA* (A) and its neighbour genes (B–D) as well as cotton orthologues (E–G) of Arabidopsis *CUC2* in normal (MCU-5) and okra leaf (Siokra 1-4) *G. hirsutum* accessions. For each gene except *GhOKRA*, primers were designed based on the D_5_ and A_2_ genome sequences to amplify both the D_t_ and A_t_ subgenome alleles. For *GhOKRA*, D_t_- and A_t_-specific primers were used, but expression was only detected in the D_t_ subgenome. Designation of the gene names was based on the BLAST search results using the corresponding D_5_ genes as queries to search against the annotated genes of the newly released *G. hirsutum* genomes (Li *et al.*, 2015; Zhang *et al.*, 2015). Genes shown in (A)–(G) are *G. hirsutum* orthologues of *Gorai.002g244000*, *Gorai.002g23900*, *Gorai.002g244100*, *Gorai.002g244200*, *Gorai.002g067300*, *Gorai.007g323900*, and *Gorai.013g171300*, respectively. For *Gorai.002g23900*, no corresponding gene was annotated in the *G. hirsutum* genome reported by Li *et al.* (2015). In the *G. hirsutum* genome reported by Zhang *et al.* (2015), the orthologue of *Gorai.002g244100* was not annotated, and *D01G2042* was found to be a combination of *Gorai.002g244000* and *Gorai.002g244200*. *Gorai.002g244200* is a homologue of *Gorai.002g244000*. Cotton ubiquitin gene (GenBank accession no. EU604080) was used as the reference. Data shown are the average of three biological replicates. Error bars represent standard deviation.

Siokra 1-4 starts to show the okra leaf phenotype at the third leaf and becomes obvious after the fourth- to fifth-leaf stage (Supplementary Fig. S1A). We reasoned that if *GhOKRA-D*_*t*_ is the gene responsible for the okra leaf, there should be a similar progression in its expression in leaves (mature and/or young) of different developmental stages. The expression profile of *GhOKRA* was therefore determined in the cotyledon and first, third, and fifth mature leaves, as well as the SAM (including young developing leaves with a length of ≤1cm) at the first-, third-, and fifth-leaf stages. No obvious difference in expression was seen in mature leaves between MCU-5 and Siokra 1-4, but in the SAM tissues, the expression levels of *GhOKRA* were higher in Siokra 1-4 than in MCU-5, and increased gradually from the first-leaf stage to the fifth-leaf stage in both MCU-5 and Siokra 1-4, but particularly in Siokra 1-4 (Fig. 4A). In the SAM at the fifth-leaf stage, the expression level of *GhOKRA* was ~3-fold higher in Siokra 1-4 than in MCU-5, consistent with the more distinct okra leaf shape of the new leaves developing at this time. In Arabidopsis leaves, *At5g03790* (*LATE MERISTEM IDENTITY1* or *LMI1*), which is closely related to *GhOKRA*, is specifically expressed in the margin of young expanding leaves (Saddic *et al.*, 2006). We found that *GhOKRA* was almost equally expressed in leaf margin and the leaf tissue flanking the midvein (leaf interior) in both MCU-5 and Siokra 1-4 (Fig. 4A).

We also analysed the expression profiles of the genes flanking *GhOKRA* in *G. hirsutum*, including orthologues of *Gorai.002G243900*, *Gorai.002G244100*, and *Gorai.002G244200* (possible paralogue of *Gorai.002G244000*). Interestingly, the *G. hirsutum* orthologue (*D01G2041/A01G1800*) of *Gorai.002G243900* had a very similar expression pattern to *GhOKRA* in all analysed tissues of both MCU-5 and Siokra 1-4 (Fig. 4B). Similar phenomena have been reported recently for the genes at the *Li*_*1*_ locus (Thyssen *et al.*, 2015). This could be a result of shared chromatin environments between these two genes as reported in Arabidopsis (Chen *et al.*, 2010). The expression level of the *G. hirsutum* orthologue (*CotAD_18409*) of *Gorai.002G244100* in MCU-5 was quite low in all analysed tissues. In Siokra 1-4, *CotAD_18409* was more highly expressed in the SAM than in other tissues and increased its expression gradually in the SAM from the first-leaf stage to the fifth-leaf stage (Fig. 4C), which is similar to the expression pattern of *GhOKRA* in Siokra 1-4 (Fig. 4A). In contrast to *GhOKRA*, its paralogue, *CotAD_18407/A01G1802*, showed no significant expression differences between MCU-5 and Siokra 1-4 in all SAM samples, instead, a gradually increasing expression level was observed from the first, to the third and fifth mature leaf of MCU-5 (Fig. 4D), suggesting a different role to *GhOKRA* in leaf development.

PINFORMED1 (PIN1) and CUP-SHAPED COTYLEDON2 (CUC2) are two key regulators of serration formation in Arabidopsis leaves (Nikovics *et al.*, 2006; Bilsborough *et al.*, 2011). *CUC2* is mainly expressed in the leaf sinus (Nikovics *et al.*, 2006). To see whether the *G. hirsutum* orthologue(s) of *CUC2* also play a role in the formation of okra leaf, we identified three potential *CUC2* orthologues in *G. raimondii* and *G. arboreum*, as well as in *G. hirsutum*, and investigated their expression in *G. hirsutum*. All three *CUC2* candidates (*D01G0448/A01G0440/CotAD_28138*, *D11G2947/A11G2580/CotAD_16876*, and *D13G1561/A11G1259/CotAD_06127*) were expressed at a relatively higher level in the SAM than in other tissues, but a gradual increase in expression in the SAM at the first-, third- and fifth-leaf stages was observed only for *D13G1561/A11G1259/CotAD_06127*, which also showed a much higher level of expression in Siokra 1-4 than in MCU-5 (Fig. 4E–G). The expression pattern of *D13G1561/A11G1259/CotAD_06127* in Siokra 1-4 was positively correlated with the development of the okra leaf trait. These results suggest that *D13G1561/A11G1259/CotAD_06127*, the closest to Arabidopsis *CUC2* out of the three cotton *CUC2* genes ( Supplementary Fig. S3), may be involved in the formation of leaf lobes in cotton.

### Possible origin of the okra leaf allele in *G. hirsutum*

The D_t_ subgenome donor of *G. hirsutum* and *G. barbadense* is thought to be a D-genome-like species very similar to modern *G. raimondii* (Wendel and Cronn, 2003). Relative to *G. raimondii* and the five *G. hirsutum* accessions showing a normal leaf phenotype, the okra/superokra leaf accessions examined had a single-nucleotide insertion (G) at position 589 of *GhOKRA-D*_*t*_ matching the same nucleotide in the A_t_ subgenome homoeologue of all varieties sequenced, irrespective of leaf shape (Fig. 3A). This insertion was confirmed by the KASP assay to be present in all other okra leaf accessions used in this study and must have originated after the formation of the allotetraploid, as it is not observed in *G. raimondii*. Because of this insertion, the coding sequence of *GhOKRA-D*_*t*_ became longer (Fig. 3B and Supplementary Fig. S6), which coincides with the leaf shape change, suggesting that the extended protein sequence may play a role in the determination of leaf shape. This was supported by the observation that a single-nucleotide deletion at position 578 in the *G. hirsutum* accession 89004-64 caused the superokra leaf phenotype (Fig. 3A and Supplementary Fig. S1B). This deletion restored the length of GhOKRA-D_t_ to the same size as that of Gorai.002G244000 but changed the C terminus of GhOKRA-D_t_ (Fig. 3B). The deletion at position 589 observed in both *G. raimondii* and all *G. hirsutum* accessions showing normal leaf shape is consistent with the notions on the origins of the D_t_ subgenome in *G. hirsutum*. GhOKRA-D_t_ of normal leaf accessions was truncated (presumably after the formation of the allotetraploid, as it does not occur in *G. raimondii*) due to the 8bp deletion so the sequences flanking position 589 would no longer be part of the GhOKRA-D_t_ protein in these accessions and hence would not be subject to positive functional selection.

The G at position 589 observed in the okra/superokra leaf *G. hirsutum* accessions could have been derived from an NRHR event, or gene conversion, between the D_t_ and A_t_ homoeologues in the ancestor of the okra leaf accessions. We noticed that the haplotype of Siokra 1-4 *GhOKRA-D*_*t*_ from position 578 to the stop codon is identical to that of *GhOKRA-A*_*t*_. An NRHR event could have occurred in the region between position 578 and the end of the sequence shown in Fig. 3A. This NRHR event would have resulted in GhOKRA-D_t_ in Siokra 1-4 being longer than Gorai.002G244000 and may have contributed to the origin of the okra leaf phenotype. The single-nucleotide deletion at position 578 observed in the superokra leaf accession 89004-64 would have occurred after the NRHR event in *GhOKRA-D*_*t*_ (Fig. 3A). In addition, the identical *GhOKRA-D*_*t*_ observed in T586 (an okra leaf marker line from the USA) and Siokra 1-4 (an okra leaf cultivar developed in Australia), and the results from phylogenetic analysis of the okra leaf accessions ( Supplementary Fig. S7 at *JXB* online) suggest a single source of the okra leaf trait and an almost identical genetic background around *GhOKRA-D*_*t*_ in the okra leaf accessions used in this study.

## Discussion

In this study, we identified *GhOKRA-D*_*t*_, which encodes an HD-Zip class I protein, as the best candidate gene determining the okra leaf trait in *G. hirsutum*. Although we have not yet carried out functional confirmation using a transgenic approach, our conclusion was supported by multiple lines of evidence. Firstly, in the *G. raimondii* genome, the region corresponding to the mapped okra leaf locus of *G. hirsutum* contains only a single gene, *Gorai.002G244000* (Fig. 1). Secondly, the closest genes of *GhOKRA-D*_*t*_ in Arabidopsis (*LMI1*) and *Cardamine hirsuta* (*REDUCED COMPLEXITY* or *RCO*) have demonstrated roles in the determination of leaf serration and leaflet formation (Saddic *et al.*, 2006; Vlad *et al.*, 2014). In addition, *cis*-regulatory variation in *RCO* contributes to the difference in leaf margin dissection observed between two sister species, *Capsella rubella* and *Capsella grandiflora* (Sicard *et al.*, 2014). Thirdly, amino acid changes caused by non-synonymous SNPs and variable C-terminal protein sequences were found in GhOKRA-D_t_ between the normal and okra leaf upland cottons, and a single-nucleotide deletion near the 3′ end of the okra leaf allele caused the superokra leaf phenotype. Fourthly, the expression levels of *GhOKRA-D*_*t*_ in the SAM sampled from different developmental stages were higher in Siokra 1-4 (okra) than in MCU-5 (normal) and correlated positively with developmental expression of the okra leaf trait (Fig. 4A and Supplementary Fig. S1). Finally, the *G. arboreum* orthologue (*A_00507*) of *GhOKRA-D*_*t*_ also co-segregated with the leaf shape trait in a *G. arboreum* F_2_ population, although it does not carry the same mutations as the D_t_ subgenome form in the tetraploid species.

Association mapping is an alternative to traditional QTL mapping, which uses the historic recombination events from many lineages to discover markers associated with or linked to genes controlling the trait (Brachi *et al.*, 2011). Here, we considered the *G. hirsutum* accessions used in the targeted association analysis as a segregating population because the okra leaf trait expressed in these lines had the same original origin. This strategy proved to be practical and powerful in view of our results, which mapped the okra leaf locus to an interval initially with only two genes that are only ~1.1kb apart. If the distance between the two genes were larger, it would have been possible to narrow down the interval to a single gene resolution. Although in total 177 *G. hirsutum* accessions were used, only 92 accessions were applied in the final fine mapping using a KASP marker assay. Obviously, this approach is much more cost effective compared with using large F_2_ segregating populations.

The okra leaf trait was mapped to the D_t_ subgenome of *G. hirsutum*, whose D_t_ and A_t_ subgenomes are thought to be similar to the extant D_5_ and A_2_ genomes, respectively (Wendel and Cronn, 2003). According to our RACE and qRT-PCR experiments, *GhOKRA-A*_*t*_ seems to be expressed at a very low level in *G. hirsutum*, if at all, suggesting loss of function or pseudogenization of *GaOKRA* (i.e. *A_00507*, the *G. arboreum* orthologue of *GhOKRA-A*_*t*_) after polyploidization, which could be a result of accumulating negative mutations in the coding or promoter regions of *GhOKRA-A*_*t*_ (Flagel and Wendel, 2009). These two situations could be distinguished by generating transgenic plants harbouring constructs of the *GhOKRA-D*_*t*_ promoter fused with *GhOKRA-A*_*t*_ or of the *GhOKRA-A*_*t*_ promoter fused with *GhOKRA-D*_*t*_. By contrast, the ancestral *Gorai.002G244000* seems to have retained its function after polyploidization and experienced a gain of function due to the non-synonymous nucleotide mutation at position 109, and changed C-terminal protein sequences in okra leaf accessions (Fig. 3) and/or promoter sequence variation between the normal and okra leaf accessions (Supplementary Fig. S8 at *JXB* online). Differential or biased expression of duplicated genes is postulated to contribute to phenotypic variation (Buggs *et al.*, 2010). In *G. hirsutum*, the extent of homoeologue expression bias and expression level dominance increases over the time from genome merger through evolution (Yoo *et al.*, 2013). Gain-of-function point mutations in homoeologous genes have also been reported in *Brassica napus* (Lu *et al.*, 2012). Interestingly, *GhOKRA-D*_*t*_ of Siokra 1-4 (okra) is identical to *GbOKRA-D*_*t*_ (the *G. barbadense* orthologue of *GhOKRA-D*_*t*_) in 3-79, Pima A8, and Sipima 280 (all subokra), suggesting that the precise functionality of the okra leaf gene is species specific. It will be useful and interesting to uncover the genetic determinant(s) underlying the species-dependent phenotypic outcomes of this same gene.

NRHR has been reported to occur throughout polyploid divergence and speciation in the genus *Gossypium*, and in *G. hirsutum*, ~1.8% of genes could have experienced NRHR since its origin ~1–2 million years ago (Salmon *et al.*, 2010). We identified a potential NRHR event in the *G. hirsutum* accessions showing the okra leaf phenotype, which occurred at the region between position 578 and the end of the sequence shown in Fig. 3A. For *G. barbadense*, the sequences of *GbOKRA-D*_*t*_ between position 578 and the end of the sequences from all three sequenced accessions were identical to that of *GhOKRA-D*_*t*_ of Siokra 1-4 (Fig. 3). In view of *G. barbadense* having the same D_t_ subgenome donor as *G. hirsutum*, it is uncertain whether the NRHR event occurred first in *G. hirsutum* and was then introgressed into *G. barbadense* or vice versa. As the NRHR was observed in all *G. barbadense* accessions and only the *G. hirsutum* accessions showing okra leaf, we prefer to propose that the NRHR event occurred first in *G. barbadense* and was then introgressed into *G. hirsutum*.

The expression level of *GhOKRA* in the SAM that includes developing young leaves and leaf primordia increased gradually with growth of the cotton plants, suggesting that transcription of *GhOKRA* is developmental stage dependent and may have a role in the establishment of plant architecture; however, a higher expression level and a more significant increase of *GhOKRA* were observed in the okra leaf accession Siokra 1-4 compared with the normal leaf accession MCU-5, which correlated positively with the development of the okra leaf trait (Fig. 4A and Supplementary Fig. S1), suggesting a direct relationship between the level of *GhOKRA* and the okra leaf phenotype. These results also suggest a repressive role for *GhOKRA* in cell proliferation in the leaf sinus region, consistent with the function of *RCO* (Vlad *et al.*, 2014). The transcriptional difference could be related to the sequence variations observed in the promoters of *GhOKRA-D*_*t*_ in Siokra 1-4 and MCU-5( Supplementary Fig. S8) or as result of feedback regulation involving the target gene(s) of GhOKRA-D_t_. It has been shown that the difference in leaf lobbing between the sister species *C. rubella* and *C. grandiflora* is related to the expression level of *RCO* in the developing lobe caused by *cis*-regulatory variation in *RCO* (Sicard *et al.*, 2014). GhOKRA is an HD-Zip protein that is composed of a homeodomain and an adjacent leucine-zipper. HD-Zip proteins bind to specific DNA sequences as homodimers or heterodimers through their Zip domains, and the absence of a Zip abolishes their binding ability (Sessa *et al.*, 1993). We found a non-synonymous SNP located at ~80 nt before the homeodomain and variable C termini between the normal (including *G. raimondii*) and okra/superokra leaf accessions. These protein sequence changes, particularly those within the C terminus, may affect the binding ability of GhOKRA to its target gene(s), because the only difference between the okra and the superokra allele is in their C-terminal sequences. The expression profiles of *GhOKRA* in MCU-5 and Siokra 1-4 and the changes in protein sequences suggest that both transcriptional regulation of *GhOKRA* and binding activity of GhOKRA may be involved in determining leaf morphology in cotton.

Previous studies have shown that the sculpting of the leaf margin in both simple and compound leaf species relies on NAC-domain transcription factors, such as *CUC2* and *GOBLET* (Nikovics *et al.*, 2006; Berger *et al.*, 2009). *CUC2* is expressed at the boundaries of incipient serrations and leaflets (Hasson *et al.*, 2011), and is a target of the microRNA miR164 (Nikovics *et al.*, 2006). When CUC2 activity was reduced because of inactivating mutation or because of miR164 overexpression, all leaves developed smoother margins in *Arabidopsis thaliana*, whereas reduced miR164 activity increased the depth of serrations and the formation of lobes in the margin of leaflets (Nikovics *et al.*, 2006). *GOBLET* is essential for the proper specification of leaflet boundaries in the developing compound leaf in tomato (Berger *et al.*, 2009). Targeted expression of *AtKRP1*, a repressor of cell division, to the sinus area of developing Arabidopsis leaves using the promoter of *CUC2* leads to local growth repression and the formation of leaves with extreme lobbing (Malinowski *et al.*, 2011). The expression profile of the *G. hirsutum* orthologue (Fig. 4G) of *CUC2* suggests that the function of the NAC-domain transcription factor in the formation of leaf lobes is most likely conserved in cotton. It is well known that species-specific regulation of class I *KNOTTED1-LIKE HOMEOBOX1* (*KNOX1*) gene expression contributes to unlobed versus lobed leaf forms (Hake *et al.*, 2004). Although KNOX1 activity in Arabidopsis is confined to, and required for, function of the SAM (Long *et al.*, 1996), many species with dissected leaves express *KNOX1* genes in both the SAM and the leaves (Hay and Tsiantis, 2010). In *G. hirsutum*, orthologues of *KNOX1* showed elevated expression levels in the SAM (including leaf primordia) of an okra leaf accession compared with a normal leaf accession (Andres *et al.*, 2014). Therefore, it seems that *GhOKRA*, the orthologues of *CUC2* and *KNOX1* are all related to the formation of the okra leaf trait. Investigating the relationship and interactions among these genes/proteins during cotton leaf development will help us understand the fundamental biological processes of cotton leaf development and to manipulate cotton leaf shape to benefit cotton production.

In summary, fine mapping accurately localized the region harbouring the okra leaf shape gene in the cotton genome. By using a combination of sequence comparisons, co-segregation analysis and gene expression analyses, we identified an HD-Zip class I transcription factor as the best candidate gene responsible for leaf shape variation in cotton. Our results provided tools for understanding the fundamental biological processes that are responsible for the cotton leaf shape variation and will help in the design of cotton plants with an ideal leaf shape for enhanced cotton production.

## Supplementary data

Supplementary data are available at *JXB* online.

**Supplementary Table S1.** **The** 177 *G. hirsutum* accessions used in SNP and KASP assays.

**Supplementary Table S2.** Primers used in this study.

**Supplementary Table S3.** KASP genotype calls.

**Supplementary Fig. S1.** Leaf morphology of various cotton accessions.

**Supplementary Fig. S2.** F_2_-based fine mapping of the okra leaf locus.

**Supplementary Fig. S3.** Phylogeny of cotton orthologues of *Arabidopsis* CUC2.

**Supplementary Fig. S4.** The *GhOKRA-Dt* cDNA from MCU-5.

**Supplementary Fig. S5.** Alignment of the coding sequences of *Gorai.002G244000* (D_5_) and its orthologues in A_2_, AD_1_, and AD_2_ genomes.

**Supplementary Fig. S6.** Alignment of the protein sequences of GhOKRA from representative accessions used in this study.

**Supplementary Fig. S7.** Phylogenetic analysis of the region around *GhOKRA-D*_*t*_ in all okra leaf accessions genotyped by the SNP chip.

**Supplementary Fig. S8.** Alignment of the promoter sequences of *GhOKRA-D*_*t*_ from MCU-5 and Siokra 1-4.

Supplementary Data
