# Supplementary material for: Integrated mapping and characterization of the gene underlying the okra leaf trait in Gossypium hirsutum L
Source: J Exp Bot. 2015 Nov 12;67(3):763–74. doi: 10.1093/jxb/erv494 (PMC4737076; doi:10.1093/jxb/erv494)
Supplement: Supplementary Data [file supp_67_3_763__index.html]

Integrated mapping and characterization of the gene underlying the okra leaf trait in Gossypium hirsutum L — Supplementary Data 

# Integrated mapping and characterization of the gene underlying the okra leaf trait in *Gossypium hirsutum* L

## Supplementary Data

Data files

- Supplementary\_fig.\_S1\_S8.pdf - Supplementary Data
- supplementary\_tables\_S1\_S3.xlsx - Supplementary Data
