# Supplementary material for: Integrated mapping and characterization of the gene underlying the okra leaf trait in Gossypium hirsutum L
Source: J Exp Bot. 2015 Nov 12;67(3):763–74. doi: 10.1093/jxb/erv494 (PMC4737076; doi:10.1093/jxb/erv494)
Supplement: Supplementary Data [file supp_erv494_Supplementary_fig._S1_S8.pdf]

Supplementary Data

Article title: **A homeodomain gene underlying the okra leaf trait in *Gossypium hirsutum* L.**
Authors: **Qian-Hao Zhu, Jian Zhang, Dexin Liu, Warwick Stiller, Dajun Liu, Zhengsheng Zhang, Danny Llewellyn, Iain Wilson**

The following Supplementary Data are available for this article:

Supplementary Table S1 177 *G. hirsutum* accessions used in SNP and KASP assays

Supplementary Table S2 Primers used in this study

Supplementary Table S3 KASP genotype calls

Supplementary Fig. S1 Leaf morphology of various cotton accessions

Supplementary Fig. S2 F_2-based fine mapping of the okra leaf locus

Supplementary Fig. S3 Phylogeny of cotton orthologues of *Arabidopsis* CUC2

Supplementary Fig. S4 The *GhOKRA-*D_t cDNA from MCU-5

Supplementary Fig. S5 Alignment of the coding sequences of *Gorai.002G244000* (D_5) and its orthologues in A_2, AD_1 and AD_2 genomes

Supplementary Fig. S6 Alignment of the protein sequences of GhOKRA from representative accessions used in this study

Supplementary Fig. S7 Phylogenetic analysis of the region around *GhOKRA-*D_t in all okra leaf accessions genotyped by the SNP chip

Supplementary Fig. S8 Alignment of the promoter sequences of *GhOKRA-*D_t from MCU-5 and Siokra 1-4

Fig. S1 Leaf morphology of various cotton accessions. (A) Leaf of MCU-5 (top panel) and Siokra 1-4 (bottom panel) at the developmental stages indicated on top. (B) Representative mature leaf of 89004-64 (super-okra; *G. hirsutum*). (C) Representative mature leaf of M18 (similar to sub-okra; *G. arboreum*).

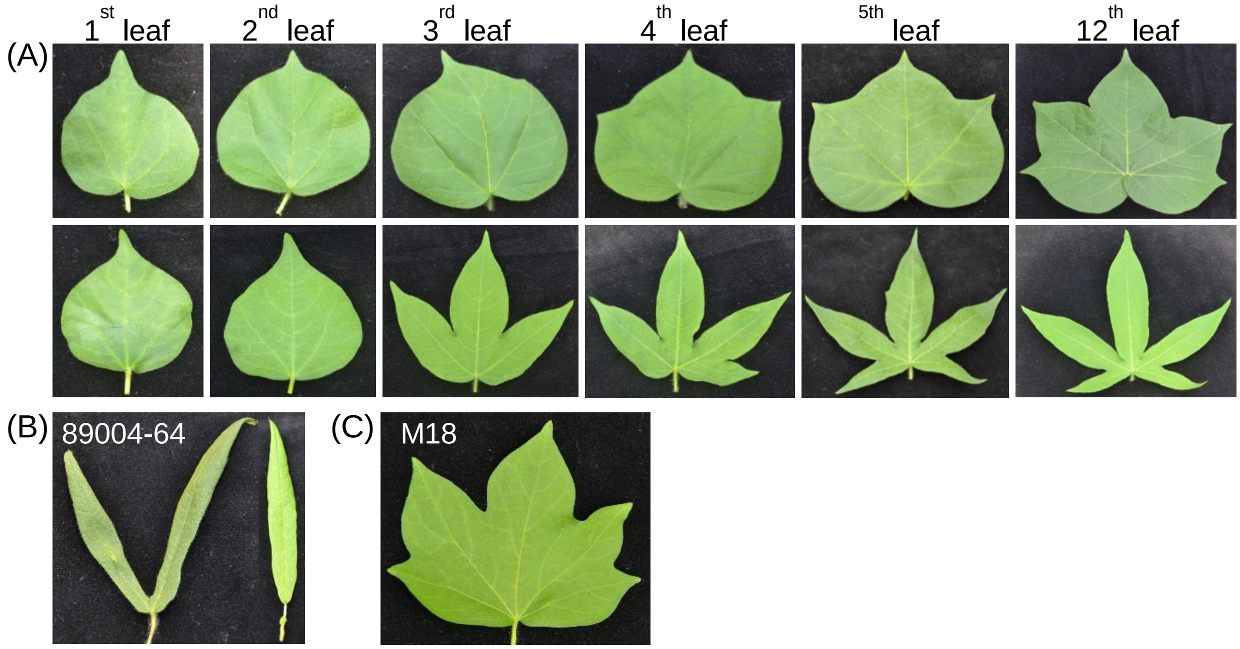

Fig. S2 F_2-based fine mapping of the okra leaf locus. (A) Genetic map of the okra leaf locus (*L2*) in *G. hirsutum* based on the RIL034 x Yumian1 F_2 population (left panel) and the corresponding physical map around the okra leaf locus in *G. raimondii* (right panel). (B) Genetic map of the okra leaf locus (*L2*) in *G. hirsutum* based on the RIL090 x Jinnong08 F_2 population (left panel) and the corresponding physical map around the okra leaf locus in *G. raimondii* (right panel). Markers showing distorted segregation are indicated by * or ***.

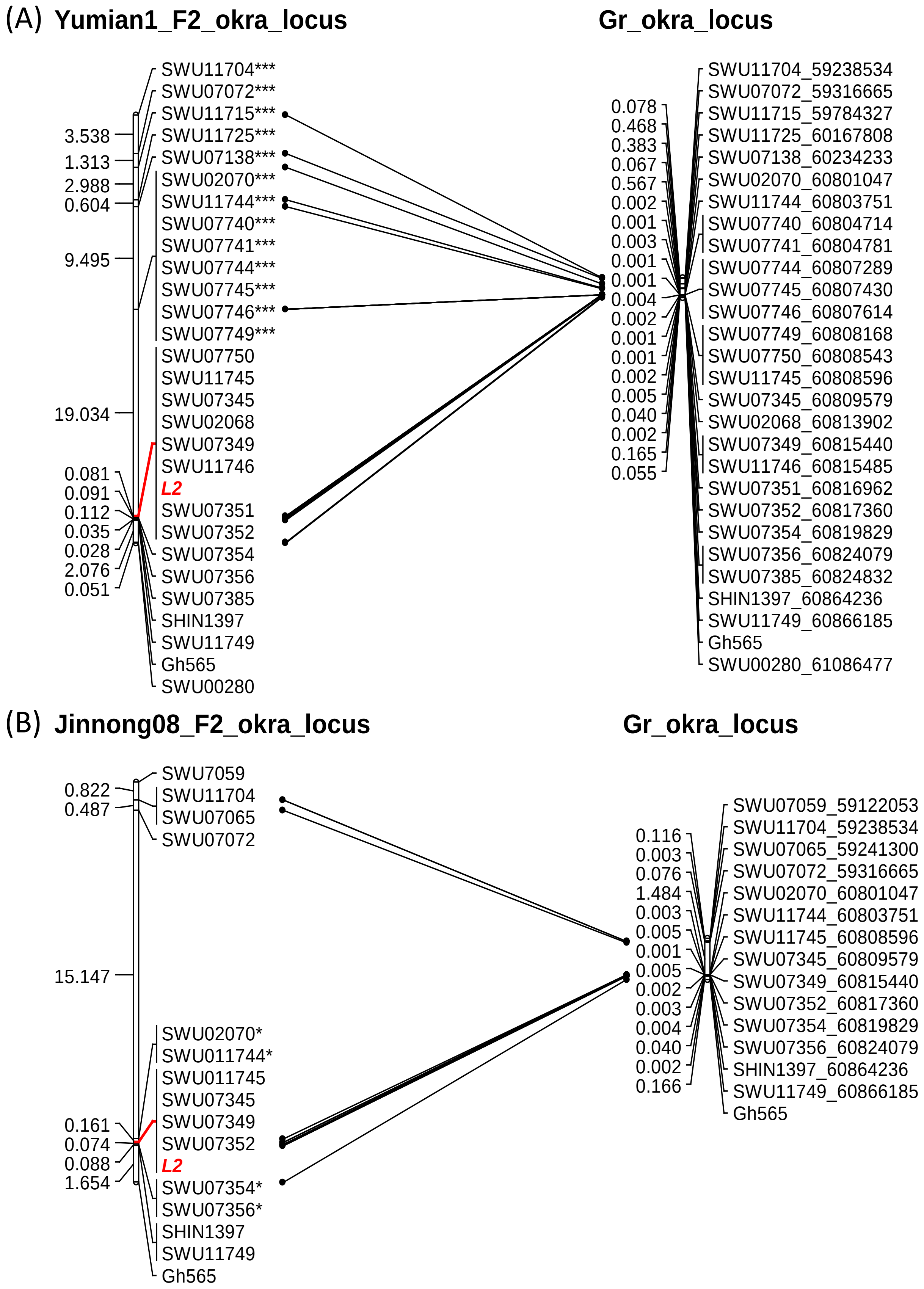

Fig. S3 Phylogeny of cotton orthologues of *Arabidopsis* CUC2. Gorai.002G067300, Gorai.007G323900 and Gorai.013G171300 are from *G. raimondii*; A_06256, A_17275 and A_16773 are from *G. arboreum*. Their orthologues in the A_t and D_t sub-genomes of *G. hirsutum* are shown after the forward slash. Annotated protein sequences were used in generating the phylogenetic tree using MEGA6 software.

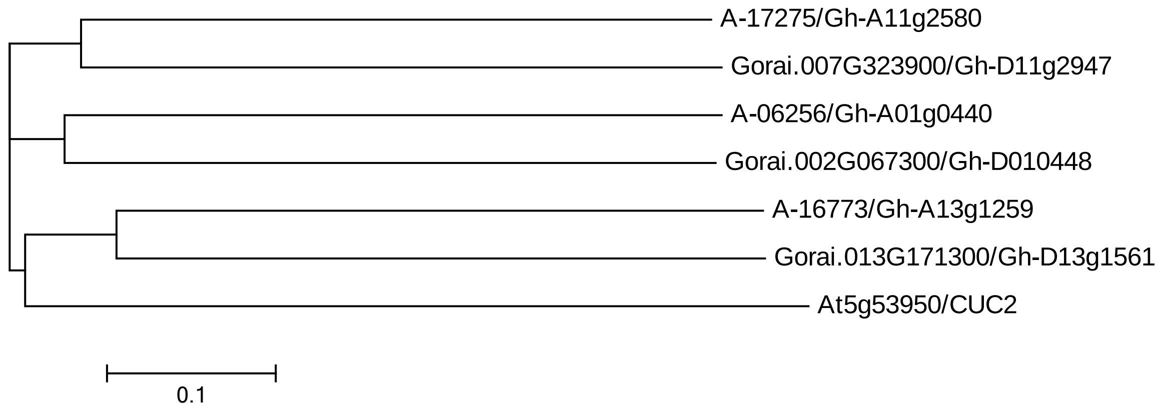

Fig. S4 The *GhOKRA*-D_t cDNA from MCU-5

GCTTCGTTCAGTTCTGGTGGTTCCATTTATATATACAGATATACTCGCTTTCTTCATTCAAAGAA**ATG**GATTGGGATGGCACC
ATTCGACCCTTTATTTCACGACTAGAACCTTCTCTTAACTTCCCTTCTAACTATAATTATAATCAATATCCAGAAGGTGTGGA
GGACATGAACAATCAAGGATTTGAAGAAGCTGGCAATGGGTTGGTTCCAGATTTGAATATGAACGGCTTCATGAACAATGGTA
AAGGTAAAAACAACAAGAAGAAGAGATTGACAAGTGATCAGTTAGATTCGTTGGAAAGGAGTTTCCAAGAAGAGAATAAGTTG
GATCCCGACAGGAAAATGAAGCTTTCCAAGGAACTTGGACTTCAACCAAGACAAATCGCTGTCTGGTTCCAAAACAGGCGTGC
TAGATGGAAAGCTAAACAGCTTGAACACTCATATAATACGCTTAAACATGAGTATGATATTATCTCCATTGAAAAGCAAAAGC
TACAAGACGAGGTGATGGAATTGAAAGGAATGCTAGGCGACAAGGAACCAAGTCTCCACGGTTTACAAGGAAATCTCCGGCGG
AGAGATCATCGAAAG**TAG**CTCGATTCGGAGCTCGAACAAGACGAGTATAGCAGGAAACGACTATATCCGATAGTTGAATGTAA
CTATATTTTCAATGAGGATGAGAATAACCCAGTTTCCACTCACTACTGGGATATTCAACTCCCTTCTTATCCCTAAAACACTA
GTTTAATTAAGCACTTAATCTTAGTTTGCTTAGTTTTTTTTAGGTTGGTCCTCGTTTCAGTACAATTTGGATTTTGGTTTGAA
GACTTTGTTGGAGAAATTGACGTAACATTAATGTATCAAAGGCTGCATCAGTGAGTATTGGATCAAAAA

Fig. S5 Alignment of the coding sequences of *Gorai.002G244000* (D_5) and its orthologues in A_2, AD_1 and AD_2 genomes. Okra and super-okra leaf accessions are highlighted in red. The causal SNP is indicated in bold red type and the predicted stop codons are underlined in bold black type.

```
Gorai.002G244000_D5         ATGGATTGGGATGGCACCATTCGACCCTTTATTTCACGACTAGAACCTTCTCTTAACTTC 60
Coker 315_Dt                ATGGATTGGGATGGCACCATTCGACCCTTTATTTCACGACTAGAACCTTCTCTTAACTTC 60
MCU-5_Dt                    ATGGATTGGGATGGCACCATTCGACCCTTTATTTCACGACTAGAACCTTCTCTTAACTTC 60
Yumian1_Dt                  ATGGATTGGGATGGCACCATTCGACCCTTTATTTCACGACTAGAACCTTCTCTTAACTTC 60
Sicot 71_Dt                 ATGGATTGGGATGGCACCATTCGACCCTTTATTTCACGACTAGAACCTTCTCTTAACTTC 60
TM-1_Dt                     ATGGATTGGGATGGCACCATTCGACCCTTTATTTCACGACTAGAACCTTCTCTTAACTTC 60
89004-64_Dt                 ATGGATTGGGATGGCACCATTCGACCCTTTATTTCACGACTAGAACCTTCTCTTAACTTC 60
Siokra 1-4_Dt               ATGGATTGGGATGGCACCATTCGACCCTTTATTTCACGACTAGAACCTTCTCTTAACTTC 60
T586_Dt                     ATGGATTGGGATGGCACCATTCGACCCTTTATTTCACGACTAGAACCTTCTCTTAACTTC 60
Sipima 280_Dt               ATGGATTGGGATGGCACCATTCGACCCTTTATTTCACGACTAGAACCTTCTCTTAACTTC 60
Pima A8_Dt                  ATGGATTGGGATGGCACCATTCGACCCTTTATTTCACGACTAGAACCTTCTCTTAACTTC 60
3-79_Dt                     ATGGATTGGGATGGCACCATTCGACCCTTTATTTCACGACTAGAACCTTCTCTTAACTTC 60
Coker 315_At                ATGGATTGGAATGGCACCATTCGACCCTTTATTTCACGACTAGAACCTTCTCTTAACTTC 60
MCU-5_At                    ATGGATTGGAATGGCACCATTCGACCCTTTATTTCACGACTAGAACCTTCTCTTAACTTC 60
Yumian1_At                  ATGGATTGGGATGGCACCATTCGACCTTTTATTTCACGACTAGAAACTTCTCTTAACTTC 60
Sicot 71_At                 ATGGATTGGAATGGCACCATTCGACCCTTTATTTCACGACTAGAACCTTCTCTTAACTTC 60
Siokra 1-4_At               ATGGATTGGAATGGCACCATTCGACCCTTTATTTCACGACTAGAACCTTCTCTTAACTTC 60
T586_At                     ATGGATTGGGATGGCACCATTCGACCCTTTATTTCACGACTAGAACCTTCTCTTAACTTC 60
Pima A8_At                  ATGGATTGGAATGGCACCATTCGACCCTTTATTTCACGACTAGAAACTTCTCTTAACTTC 60
3-79_At                     ATGGATTGGAATGGCACCATTCGACCCTTTATTTCACGACTAGAAACTTCTCTTAACTTC 60
A-00507_A2                  ATGGATTGGAATGGCACCATTCGACCCTTTATTTCACGACTAGAACCTTCTCTTAACTTC 60
YZ                          ATGGATTGGAATGGCACCATTCGACCCTTTATTTCACGACTAGAACCTTCTCTTAACTTC 60
M18                         ATGGATTGGAATGGCACCATTCGACCCTTTATTTCACGACTAGAACCTTCTCTTAACTTC 60
BM13H                       ATGGATTGGAATGGCACCATTCGACCCTTTATTTCACGACTAGAACCTTCTCTTAACTTC 60
                            ********* **************** ****************** **************

Gorai.002G244000_D5         CCTTCTAACTATAATTATAATCAATATCCAGAAGGTGTGGAGGACATGAACAATCAAGGA 120
Coker 315_Dt                CCTTCTAACTATAATTATAATCAATATCCAGAAGGTGTGGAGGACATGAACAATCAAGGA 120
MCU-5_Dt                    CCTTCTAACTATAATTATAATCAATATCCAGAAGGTGTGGAGGACATGAACAATCAAGGA 120
Yumian1_Dt                  CCTTCTAACTATAATTATAATCAATATCCAGAAGGTGTGGAGGACATGAACAATCAAGGA 120
Sicot 71_Dt                 CCTTCTAACTATAATTATAATCAATATCCAGAAGGTGTGGAGGACATGAACAATCAAGGA 120
TM-1_Dt                     CCTTCTAACTATAATTATAATCAATATCCAGAAGGTGTGGAGGACATGAACAATCAAGGA 120
89004-64_Dt                 CCTTCTAACTATAATTATAATCAATATCCAGAAGGTGTGGAGGACATGGACAATCAAGGA 120
Siokra 1-4_Dt               CCTTCTAACTATAATTATAATCAATATCCAGAAGGTGTGGAGGACATGGACAATCAAGGA 120
T586_Dt                     CCTTCTAACTATAATTATAATCAATATCCAGAAGGTGTGGAGGACATGGACAATCAAGGA 120
Sipima 280_Dt               CCTTCTAACTATAATTATAATCAATATCCAGAAGGTGTGGAGGACATGGACAATCAAGGA 120
Pima A8_Dt                  CCTTCTAACTATAATTATAATCAATATCCAGAAGGTGTGGAGGACATGGACAATCAAGGA 120
3-79_Dt                     CCTTCTAACTATAATTATAATCAATATCCAGAAGGTGTGGAGGACATGGACAATCAAGGA 120
Coker 315_At                CCTTATAACTATAATTATAATCAATATCCAG---GTGTGGAGGACATGAACAATCAAGGA 117
MCU-5_At                    CCTTATAACTATAATTATAATCAATATCCAG---GTGTGGAGGACATGAACAATCAAGGA 117
Yumian1_At                  CCTTATAACTATAATTATAATCAATATCCAG---GTGTGGAGGACATGAACAATCAAGGA 117
Sicot 71_At                 CCTTATAACTATAATTATAATCAATATCCAG---GTGTGGAGGACATGAACAATCAAGGA 117
Siokra 1-4_At               CCTTATAACTATAATTATAATCAATATCCAG---GTGTGGAGGACATGAACAATCAAGGA 117
T586_At                     CCTTATAACTATAATTATAATCAATATCCAG---GTGTGGAGGACATGAACAATCAAGGA 117
Pima A8_At                  CCTTATAACTATAATTATAATCAATATCCAG---GTGTGGAGGACATGAACAATCAAGGA 117
3-79_At                     CCTTATAACTATAATTATAATCAATATCCAG---GTGTGGAGGACATGAACAATCAAGGA 117
A-00507_A2                  CCTTATAACTATAATTATAATCAATATCCAG---GTGTGGAGGACATGAACAATCAAGGA 117
YZ                          CCTTATAACTATAATTATAATCAATATCCAG---GTGTGGAGGACATGAACAATCAAGGA 117
M18                         CCTTATAACTATAATTATAATCAATATCCAG---GTGTGGAGGACATGAACAATCAAGGA 117
BM13H                       CCTTATAACTATAATTATAATCAATATCCAG---GTGTGGAGGACATGAACAATCAAGGA 117
                            ** * **************************   ************** ***********

Gorai.002G244000_D5         TTTGAAGAAGCTGGCAATGGGTTGGTTCCAGATTTGAATATGAACGGCTTCATGAACAAT 180
Coker 315_Dt                TTTGAAGAAGCTGGCAATGGGTTGGTTCCAGATTTGAATATGAACGGCTTCATGAACAAT 180
MCU-5_Dt                    TTTGAAGAAGCTGGCAATGGGTTGGTTCCAGATTTGAATATGAACGGCTTCATGAACAAT 180
Yumian1_Dt                  TTTGAAGAAGCTGGCAATGGGTTGGTTCCAGATTTGAATATGAACGGCTTCATGAACAAT 180
Sicot 71_Dt                 TTTGAAGAAGCTGGCAATGGGTTGGTTCCAGATTTGAATATGAACGGCTTCATGAACAAT 180
TM-1_Dt                     TTTGAAGAAGCTGGCAATGGGTTGGTTCCAGATTTGAATATGAACGGCTTCATGAACAAT 180
89004-64_Dt                 TTTGAAGAAGCTGGCAATGGGTTGGTTCCAGATTTGAATATGAACGGCTTCATGAACAAT 180
Siokra 1-4_Dt               TTTGAAGAAGCTGGCAATGGGTTGGTTCCAGATTTGAATATGAACGGCTTCATGAACAAT 180
T586_Dt                     TTTGAAGAAGCTGGCAATGGGTTGGTTCCAGATTTGAATATGAACGGCTTCATGAACAAT 180
Sipima 280_Dt               TTTGAAGAAGCTGGCAATGGGTTGGTTCCAGATTTGAATATGAACGGCTTCATGAACAAT 180
Pima A8_Dt                  TTTGAAGAAGCTGGCAATGGGTTGGTTCCAGATTTGAATATGAACGGCTTCATGAACAAT 180
3-79_Dt                     TTTGAAGAAGCTGGCAATGGGTTGGTTCCAGATTTGAATATGAACGGCTTCATGAACAAT 180
Coker 315_At                TTTGAAGAAGCTGGCAATGGGTTGGTTCCAGATTTGAATATGAACAGCTTCAAGAACAAT 177
MCU-5_At                    TTTGAAGAAGCTGGCAATGGGTTGGTTCCAGATTTGAATATGAACAGCTTCAAGAACAAT 177
Yumian1_At                  TTTGAAGAAGCTGGCAATGGGTTGGTTCCAGATTTGAATATGAACAGCTTCAAGAACAAT 177
Sicot 71_At                 TTTGAAGAAGCTGGCAATGGGTTGGTTCCAGATTTGAATATGAACAGCTTCAAGAACAAT 177
Siokra 1-4_At               TTTGAAGAAGCTGGCAATGGGTTGGTTCCAGATTTGAATATGAACAGCTTCAAGAACAAT 177
T586_At                     TTTGAAGAAGCTGGCAATGGGTTGGTTCCAGATTTGAATATGAACAGCTTCAAGAACAAT 177
Pima A8_At                  TTTGAAGAAGCTGGCAATGGGTTGGTTCCAGATTTGAATATGAACAGCTTCAAGAACAAT 177
3-79_At                     TTTGAAGAAGCTGGCAATGGGTTGGTTCCAGATTTGAATATGAACAGCTTCAAGAACAAT 177
A-00507_A2                  TTTGAAGAAGCTGGCAATGGGTTGGTTCCAGATTTGAATATGAACAGCTTCAAGAACAAT 177
YZ                          TTTGAAGAAGCTGGCAATGGGTTGGTTCCAGATTTGAATATGAACAGCTTCAAGAACAAT 177
M18                         TTTGAAGAAGCTGGCAATGGGTTGGTTCCAGATTTGAATATGAACAGCTTCAAGAACAAT 177
BM13H                       TTTGAAGAAGCTGGCAATGGGTTGGTTCCAGATTTGAATATGAACAGCTTCAAGAACAAT 177
                            **********************************      ***** ****** *******
```

```
Gorai.002G244000_D5      GGTAAAGGTAACAACAACAAGAAGAAGAGATTGACAAGTGATCAGTTAGATTCGTTGGAA 240
Coker 315_Dt             GGTAAAGGTAAAAACAACAAGAAGAAGAGATTGACAAGTGATCAGTTAGATTCGTTGGAA 240
MCU-5_Dt                 GGTAAAGGTAAAAACAACAAGAAGAAGAGATTGACAAGTGATCAGTTAGATTCGTTGGAA 240
Yumian1_Dt               GGTAAAGGTAAAAACAACAAGAAGAAGAGATTGACAAGTGATCAGTTAGATTCGTTGGAA 240
Sicot 71_Dt              GGTAAAGGTAAAAACAACAAGAAGAAGAGATTGACAAGTGATCAGTTAGATTCGTTGGAA 240
TM-1_Dt                  GGTAAAGGTAAAAACAACAAGAAGAAGAGATTGACAAGTGATCAGTTAGATTCGTTGGAA 240
89004-64_Dt              GGTAAAGGTAACAACAACAAGAAGAAGAGATTGACAAGTGATCAGTTAGATTCGTTGGAA 240
Siokra 1-4_Dt            GGTAAAGGTAACAACAACAAGAAGAAGAGATTGACAAGTGATCAGTTAGATTCGTTGGAA 240
T586_Dt                  GGTAAAGGTAACAACAACAAGAAGAAGAGATTGACAAGTGATCAGTTAGATTCGTTGGAA 240
Sipima 280_Dt            GGTAAAGGTAACAACAACAAGAAGAAGAGATTGACAAGTGATCAGTTAGATTCGTTGGAA 240
Pima A8_Dt               GGTAAAGGTAACAACAACAAGAAGAAGAGATTGACAAGTGATCAGTTAGATTCGTTGGAA 240
3-79_Dt                  GGTAAAGGTAACAACAACAAGAAGAAGAGATTGACAAGTGATCAGTTAGATTCGTTGGAA 240
Coker 315_At             GGTAAAGGTAACAACAAGAAGAAGAAGAGGTTGACAAGTGATCAGTTAGATTCGTTGGAA 237
MCU-5_At                 GGTAAAGGTAACAACAAGAAGAAGAAGAGGTTGACAAGTGATCAGTTAGATTCGTTGGAA 237
Yumian1_At               GGTAAAGTTAACAACAAGAAGAAGAAGAGGTTGACAAGTGATCAGTTAGATTCGTTGGAA 237
Sicot 71_At              GGTAAAGGTAACAACAAGAAGAAGAAGAGGTTGACAAGTGATCAGTTAGATTCGTTGGAA 237
Siokra 1-4_At            GGTAAAGGTAACAACAAGAAGAAGAAGAGGTTGACAAGTGATCAGTTAGATTCGTTGGAA 237
T586_At                  GGTAAAGGTAACAACAAGAAGAAGAAGAGGTTGACAAGTGATCAGTTAGATTCGTTGGAA 237
Pima A8_At               GGTAAAGTTAACAACAAGAAGAAGAAGAGGTTGACAAGTGATCAGTTAGATTCGTTGGAA 237
3-79_At                  GGTAAAGTTAACAACAAGAAGAAGAAGAGGTTGACAAGTGATCAGTTAGATTCGTTGGAA 237
A-00507_A2               GGTAAAGGTAACAACAAGAAGAAGAAGAGGTTGACAAGTGATCAGTTAGATTCGTTGGAA 237
YZ                       GGTAAAGGTAACAACAAGAAGAAGAAGAGGTTGACAAGTGATCAGTTAGATTCGTTGGAA 237
M18                      GGTAAAGGTAACAACAAGAAGAAGAAGAGGTTGACAAGTGATCAGTTAGATTCGTTGGAA 237
BM13H                    GGTAAAGGTAACAACAAGAAGAAGAAGAGGTTGACAAGTGATCAGTTAGATTCGTTGGAA 237
                         ******* *** ***** *********** ******************************

Gorai.002G244000_D5      AGGAGTTTCCAAGAAGAGAATAAGTTGGATCCCGACAGGAAAATGAAGCTTTCCAAGGAA 300
Coker 315_Dt             AGGAGTTTCCAAGAAGAGAATAAGTTGGATCCCGACAGGAAAATGAAGCTTTCCAAGGAA 300
MCU-5_Dt                 AGGAGTTTCCAAGAAGAGAATAAGTTGGATCCCGACAGGAAAATGAAGCTTTCCAAGGAA 300
Yumian1_Dt               AGGAGTTTCCAAGAAGAGAATAAGTTGGATCCCGACAGGAAAATGAAGCTTTCCAAGGAA 300
Sicot 71_Dt              AGGAGTTTCCAAGAAGAGAATAAGTTGGATCCCGACAGGAAAATGAAGCTTTCCAAGGAA 300
TM-1_Dt                  AGGAGTTTCCAAGAAGAGAATAAGTTGGATCCCGACAGGAAAATGAAGCTTTCCAAGGAA 300
89004-64_Dt              AGGAGTTTCCAAGAAGAGAATAAGTTGGATCCCGACAGGAAAATGAAGCTTTCCAAGGAA 300
Siokra 1-4_Dt            AGGAGTTTCCAAGAAGAGAATAAGTTGGATCCCGACAGGAAAATGAAGCTTTCCAAGGAA 300
T586_Dt                  AGGAGTTTCCAAGAAGAGAATAAGTTGGATCCCGACAGGAAAATGAAGCTTTCCAAGGAA 300
Sipima 280_Dt            AGGAGTTTCCAAGAAGAGAATAAGTTGGATCCCGACAGGAAAATGAAGCTTTCCAAGGAA 300
Pima A8_Dt               AGGAGTTTCCAAGAAGAGAATAAGTTGGATCCCGACAGGAAAATGAAGCTTTCCAAGGAA 300
3-79_Dt                  AGGAGTTTCCAAGAAGAGAATAAGTTGGATCCCGACAGGAAAATGAAGCTTTCCAAGGAA 300
Coker 315_At             AGGAGTTTCCAAGAAGAGAATAAGTTGGATCCCGACAGGAAAATGAAGCTTTCCAAGGAA 297
MCU-5_At                 AGGAGTTTCCAAGAAGAGAATAAGTTGGATCCCGACAGGAAAATGAAGCTTTCCAAGGAA 297
Yumian1_At               AGGAGTTTCCAAGAAGAGAATAAGTTGGATCCCGACAGGAAAATGAAGCTTTCCAAGGAA 297
Sicot 71_At              AGGAGTTTCCAAGAAGAGAATAAGTTGGATCCCGACAGGAAAATGAAGCTTTCCAAGGAA 297
Siokra 1-4_At            AGGAGTTTCCAAGAAGAGAATAAGTTGGATCCCGACAGGAAAATGAAGCTTTCCAAGGAA 297
T586_At                  AGGAGTTTCCAAGAAGAGAATAAGTTGGATCCCGACAGGAAAATGAAGCTTTCCAAGGAA 297
Pima A8_At               AGGAGTTTCCAAGAAGAGAATAAGTTGGATCCCGACAGGAAAATGAAGCTTTCCAAGGAA 297
3-79_At                  AGGAGTTTCCAAGAAGAGAATAAGTTGGATCCCGACAGGAAAATGAAGCTTTCCAAGGAA 297
A-00507_A2               AGGAGTTTCCAAGAAGAGAATAAGTTGGATCCCGACAGGAAAATGAAGCTTTCCAAGGAA 297
YZ                       AGGAGTTTCCAAGAAGAGAATAAGTTGGATCCCGACAGGAAAATGAAGCTTTCCAAGGAA 297
M18                      AGGAGTTTCCAAGAAGAGAATAAGTTGGATCCCGACAGGAAAATGAAGCTTTCCAAGGAA 297
BM13H                    AGGAGTTTCCAAGAAGAGAATAAGTTGGATCCCGACAGGAAAATGAAGCTTTCCAAGGAA 297
                         ************************************************************

Gorai.002G244000_D5      CTTGGACTTCAACCAAGACAAATCGCTGTCTGGTTCCAAAACAGGCGTGCTAGATGGAAA 360
Coker 315_Dt             CTTGGACTTCAACCAAGACAAATCGCTGTCTGGTTCCAAAACAGGCGTGCTAGATGGAAA 360
MCU-5_Dt                 CTTGGACTTCAACCAAGACAAATCGCTGTCTGGTTCCAAAACAGGCGTGCTAGATGGAAA 360
Yumian1_Dt               CTTGGACTTCAACCAAGACAAATCGCTGTCTGGTTCCAAAACAGGCGTGCTAGATGGAAA 360
Sicot 71_Dt              CTTGGACTTCAACCAAGACAAATCGCTGTCTGGTTCCAAAACAGGCGTGCTAGATGGAAA 360
TM-1_Dt                  CTTGGACTTCAACCAAGACAAATCGCTGTCTGGTTCCAAAACAGGCGTGCTAGATGGAAA 360
89004-64_Dt              CTTGGACTTCAACCAAGACAAATCGCTGTCTGGTTCCAAAACAGGCGTGCTAGATGGAAA 360
Siokra 1-4_Dt            CTTGGACTTCAACCAAGACAAATCGCTGTCTGGTTCCAAAACAGGCGTGCTAGATGGAAA 360
T586_Dt                  CTTGGACTTCAACCAAGACAAATCGCTGTCTGGTTCCAAAACAGGCGTGCTAGATGGAAA 360
Sipima 280_Dt            CTTGGACTTCAACCAAGACAAATCGCTGTCTGGTTCCAAAACAGGCGTGCTAGATGGAAA 360
Pima A8_Dt               CTTGGACTTCAACCAAGACAAATCGCTGTCTGGTTCCAAAACAGGCGTGCTAGATGGAAA 360
3-79_Dt                  CTTGGACTTCAACCAAGACAAATCGCTGTCTGGTTCCAAAACAGGCGTGCTAGATGGAAA 360
Coker 315_At             CTTGGACTTCAACCAAGACAAATCGCTGTCTGGTTCCAAAACAGGCGTGCTAGATGGAAA 357
MCU-5_At                 CTTGGACTTCAACCAAGACAAATCGCTGTCTGGTTCCAAAACAGGCGTGCTAGATGGAAA 357
Yumian1_At               CTTGGACTTCAACCAAGACAAATCGCTGTCTGGTTCCAAAACAGGCGTGCTAGATGGAAA 357
Sicot 71_At              CTTGGACTTCAACCAAGACAAATCGCTGTCTGGTTCCAAAACAGGCGTGCTAGATGGAAA 357
Siokra 1-4_At            CTTGGACTTCAACCAAGACAAATCGCTGTCTGGTTCCAAAACAGGCGTGCTAGATGGAAA 357
T586_At                  CTTGGACTTCAACCAAGACAAATCGCTGTCTGGTTCCAAAACAGGCGTGCTAGATGGAAA 357
Pima A8_At               CTTGGACTTCAACCAAGACAAATCGCTGTCTGGTTCCAAAACAGGCGTGCTAGATGGAAA 357
3-79_At                  CTTGGACTTCAACCAAGACAAATCGCTGTCTGGTTCCAAAACAGGCGTGCTAGATGGAAA 357
A-00507_A2               CTTGGACTTCAACCAAGACAAATCGCTGTCTGGTTCCAAAACAGGCGTGCTAGATGGAAA 357
YZ                       CTTGGACTTCAACCAAGACAAATCGCTGTCTGGTTCCAAAACAGGCGTGCTAGATGGAAA 357
M18                      CTTGGACTTCAACCAAGACAAATCGCTGTCTGGTTCCAAAACAGGCGTGCTAGATGGAAA 357
BM13H                    CTTGGACTTCAACCAAGACAAATCGCTGTCTGGTTCCAAAACAGGCGTGCTAGATGGAAA 357
                         ************************************************************
```

```
Gorai.002G244000_D5      GCTAAACAGCTTGAACACTCATATAATACGCTTAAACATGAGTATGATATTATCTCCATT 420
Coker 315_Dt             GCTAAACAGCTTGAACACTCATATAATACGCTTAAACATGAGTATGATATTATCTCCATT 420
MCU-5_Dt                 GCTAAACAGCTTGAACACTCATATAATACGCTTAAACATGAGTATGATATTATCTCCATT 420
Yumian1_Dt               GCTAAACAGCTTGAACACTCATATAATACGCTTAAACATGAGTATGATATTATCTCCATT 420
Sicot 71_Dt              GCTAAACAGCTTGAACACTCATATAATACGCTTAAACATGAGTATGATATTATCTCCATT 420
TM-1_Dt                  GCTAAACAGCTTGAACACTCATATAATACGCTTAAACATGAGTATGATATTATCTCCATT 420
89004-64_Dt              GCTAAACAGCTTGAACACTCATATAATACGCTTAAACATGAGTATGATATTATCTCCATT 420
Siokra 1-4_Dt            GCTAAACAGCTTGAACACTCATATAATACGCTTAAACATGAGTATGATATTATCTCCATT 420
T586_Dt                  GCTAAACAGCTTGAACACTCATATAATACGCTTAAACATGAGTATGATATTATCTCCATT 420
Sipima 280_Dt            GCTAAACAGCTTGAACACTCATATAATACGCTTAAACATGAGTATGATATTATCTCCATT 420
Pima A8_Dt               GCTAAACAGCTTGAACACTCATATAATACGCTTAAACATGAGTATGATATTATCTCCATT 420
3-79_Dt                  GCTAAACAGCTTGAACACTCATATAATACGCTTAAACATGAGTATGATATTATCTCCATT 420
Coker 315_At             GCTAAACAGCTTCAACACTCATATAATACGCTTAAACATGAGTATGATGTTACGTATATG 417
MCU-5_At                 GCTAAACAGCTTCAACACTCATATAATACGCTTAAACATGAGTATGATGTTACGTATATG 417
Yumian1_At               GCTAAACAGCTTCAACACTCATATAATACGCTTAAACATGAGTATGATGTTACGTATATG 417
Sicot 71_At              GCTAAACAGCTTCAACACTCATATAATACGCTTAAACATGAGTATGATGTTACGTATATG 417
Siokra 1-4_At            GCTAAACAGCTTCAACACTCATATAATACGCTTAAACATGAGTATGATGTTACGTATATG 417
T586_At                  GCTAAACAGCTTCAACACTCATATAATACGCTTAAACATGAGTATGATGTTACGTATATG 417
Pima A8_At               GCTAAACAGCTTCAACACTCATATAATACGCTTAAACATGAGTATGATGTTACGTATATG 417
3-79_At                  GCTAAACAGCTTCAACACTCATATAATACGCTTAAACATGAGTATGATGTTACGTATATG 417
A-00507_A2               GCTAAACAGCTTCAACACTCATATAATACGCTTAAACATGAGTATGATGTTATCTATATG 417
YZ                       GCTAAACAGCTTCAACACTCATATAATACGCTTAAACATGAGTATGATGTTATCTATATG 417
M18                      GCTAAACAGCTTCAACACTCATATAATACGCTTAAACATGAGTATGATGTTATCTATATG 417
BM13H                    GCTAAACAGCTTCAACACTCATATAATACGCTTAAACATGAGTATGATGTTATCTATATG 417
                         ************ **** ****************************** ***  *  **

Gorai.002G244000_D5      GAAAAGCAAAAGCTACAAGACGAGGTGATGGAATTGAAAGGAATGCTAGTGGAACAGGCG 480
Coker 315_Dt             GAAAAGCAAAAGCTACAAGACGAGGTGATGGAATTGAAAGGAATGCTA--------GGCG 472
MCU-5_Dt                 GAAAAGCAAAAGCTACAAGACGAGGTGATGGAATTGAAAGGAATGCTA--------GGCG 472
Yumian1_Dt               GAAAAGCAAAAGCTACAAGACGAGGTGATGGAATTGAAAGGAATGCTA--------GGCG 472
Sicot 71_Dt              GAAAAGCAAAAGCTACAAGACGAGGTGATGGAATTGAAAGGAATGCTA--------GGCG 472
TM-1_Dt                  GAAAAGCAAAAGCTACAAGACGAGGTGATGGAATTGAAAGGAATGCTA--------GGCG 472
89004-64_Dt              GAAAAGCAAAAGCTACAAGACGAGGTGATGGAATTGAAAGGAATGCTAGTGGAACAGGCG 480
Siokra 1-4_Dt            GAAAAGCAAAAGCTACAAGACGAGGTGATGGAATTGAAAGGAATGCTAGTGGAACAGGCG 480
T586_Dt                  GAAAAGCAAAAGCTACAAGACGAGGTGATGGAATTGAAAGGAATGCTAGTGGAACAGGCG 480
Sipima 280_Dt            GAAAAGCAAAAGCTACAAGACGAGGTGATGGAATTGAAAGGAATGCTAGTGGAACAGGCG 480
Pima A8_Dt               GAAAAGCAAAAGCTACAAGACGAGGTGATGGAATTGAAAGGAATGCTAGTGGAACAGGCG 480
3-79_Dt                  GAAAAGCAAAAGCTACAAGACGAGGTGATGGAATTGAAAGGAATGCTAGTGGAACAGGCG 480
Coker 315_At             GAAAAGCAAATGCTACAAGACGAGGTGATGGAATTGAAAGGAATGTTAATGGAACAGGCG 477
MCU-5_At                 GAAAAGCAAATGCTACAAGACGAGGTGATGGAATTGAAAGGAATGTTAATGGAACAGGCG 477
Yumian1_At               GAAAAGCAAATGCTACAAGACGAGGTGATGGAATTGAAAGGAATGCTAATGGAACAGGCG 477
Sicot 71_At              GAAAAGCAAATGCTACAAGACGAGGTGATGGAATTGAAAGGAATGCTAATGGAACAGGCG 477
Siokra 1-4_At            GAAAAGCAAATGCTACAAGACGAGGTGATGGAATTGAAAGGAATGCTAATGGAACAGGCG 477
T586_At                  GAAAAGCAAATGCTACAAGACGAGGTGATGGAATTGAAAGGAATGTTAATGGAACAGGCG 477
Pima A8_At               GAAAAGCAAATGCTACAAGACGAGGTGATGGAATTGAAAGGAATGCTAATGGAACAGGCG 477
3-79_At                  GAAAAGCAAATGCTACAAGACGAGGTGATGGAATTGAAAGGAATGCTAATGGAACAGGCG 477
A-00507_A2               GAAAAGCAAATGCTACAAGACGAGGTGATGGAATTGAAAGGAATGCTAATGGAACAGGCG 477
YZ                       GAAAAGCAAATGCTACAAGACGAGGTGATGGAATTGAAAGGAATGCTAATGGAACAGGCG 477
M18                      GAAAAGCAAATGCTACAAGACGAGGTGATGGAATTGAAAGGAATGCTAATGGAACAGGCG 477
BM13H                    GAAAAGCAAATGCTACAAGACGAGGTGATGGAATTGAAAGGAATGCTAATGGAACAGGCG 477
                         ********** ********************************** **        ****

Gorai.002G244000_D5      ACAAGGAACCAAGTCTCCACGGTTTACAAGGAAATCTCCGGCGGAGAGATCATCGAAAGT 540
Coker 315_Dt             ACAAGGAACCAAGTCTCCACGGTTTACAAGGAAATCTCCGGCGGAGAGATCATCGAAAGT 532
MCU-5_Dt                 ACAAGGAACCAAGTCTCCACGGTTTACAAGGAAATCTCCGGCGGAGAGATCATCGAAAGT 532
Yumian1_Dt               ACAAGGAACCAAGTCTCCACGGTTTACAAGGAAATCTCCGGCGGAGAGATCATCGAAAGT 532
Sicot 71_Dt              ACAAGGAACCAAGTCTCCACGGTTTACAAGGAAATCTCCGGCGGAGAGATCATCGAAAGT 532
TM-1_Dt                  ACAAGGAACCAAGTCTCCACGGTTTACAAGGAAATCTCCGGCGGAGAGATCATCGAAAGT 532
89004-64_Dt              ACAAGGAACCAAGTCTCCACGGTTTACAAGGAAATCTCCGGCGGAGAGATCATCGAAAGT 540
Siokra 1-4_Dt            ACAAGGAACCAAGTCTCCACGGTTTACAAGGAAATCTCCGGCGGAGAGATCATCGAAAGT 540
T586_Dt                  ACAAGGAACCAAGTCTCCACGGTTTACAAGGAAATCTCCGGCGGAGAGATCATCGAAAGT 540
Sipima 280_Dt            ACAAGGAACCAAGTCTCCACGGTTTACAAGGAAATCTCCGGCGGAGAGATCATCGAAAGT 540
Pima A8_Dt               ACAAGGAACCAAGTCTCCACGGTTTACAAGGAAATCTCCGGCGGAGAGATCATCGAAAGT 540
3-79_Dt                  ACAAGGAACCAAGTCTCCACGGTTTACAAGGAAATCTCCGGCGGAGAGATCATCGAAAGT 540
Coker 315_At             ACAAGGAACCAAGTCTCCACGGTTTACAAGGAAATCTCCGGCGGAGAGACCATCGAAAGT 537
MCU-5_At                 ACAAGGAACCAAGTCTCCACGGTTTACAAGGAAATCTCCGGCGGAGAGACCATCGAAAGT 537
Yumian1_At               ACAAGGAACCAAGTCTCCACGGTTTACAAGGAAATCTCCGGCGGAGAGACCATCGAAAGT 537
Sicot 71_At              ACAAGGAACCAAGTCTCCACGGTTTACAAGGAAATCTCCGGCGGAGAGACCATCGAAAGT 537
Siokra 1-4_At            ACAAGGAACCAAGTCTCCACGGTTTACAAGGAAATCTCCGGCGGAGAGACCATCGAAAGT 537
T586_At                  ACAAGGAACCAAGTCTCCACGGTTTACAAGGAAATCTCCGGCGGAGAGACCATCGAAAGT 537
Pima A8_At               ACAAGGAACCAAGTCTCCACGGTTTACAAGGAAATCTCCGGCGGAGAGACCATCGAAAGT 537
3-79_At                  ACAAGGAACCAAGTCTCCACGGTTTACAAGGAAATCTCCGGCGGAGAGACCATCGAAAGT 537
A-00507_A2               ACAAGGAACCAAGTCTCCACGGTTTACAAGGAAATCTCCGGCGGAGAGACCATCGAAAGT 537
YZ                       ACAAGGAACCAAGTCTCCACGGTTTACAAGGAAATCTCCGGCGGAGAGACCATCGAAAGT 537
M18                      ACAAGGAACCAAGTCTCCACGGTTTACAAGGAAATCTCCGGCGGAGAGACCATCGAAAGT 537
BM13H                    ACAAGGAACCAAGTCTCCACGGTTTACAAGGAAATCTCCGGCGGAGAGACCATCGAAAGT 537
                         ********************************************* *** **********
```

```
Gorai.002G244000_D5     AGCTCGATTCGGAGCTCGAACAAGACGAGTATAGCAGGAAACGACTAT-ATCCGATAGTT 599
Coker 315_Dt            AGCTCGATTCGGAGCTCGAACAAGACGAGTATAGCAGGAAACGACTAT-ATCCGATAGTT 591
MCU-5_Dt                AGCTCGATTCGGAGCTCGAACAAGACGAGTATAGCAGGAAACGACTAT-ATCCGATAGTT 591
Yumian1_Dt              AGCTCGATTCGGAGCTCGAACAAGACGAGTATAGCAGGAAACGACTAT-ATCCGATAGTT 591
Sicot 71_Dt             AGCTCGATTCGGAGCTCGAACAAGACGAGTATAGCAGGAAACGACTAT-ATCCGATAGTT 591
TM-1_Dt                 AGCTCGATTCGGAGCTCGAACAAGACGAGTATAGCAGGAAACGACTAT-ATCCGATAGTT 591
89004-64_Dt             AGCTCGATTCGGAGCTCGAACAAGACGAGTATAGCAG-AAACGACTATGATCCGATAGTT 599
Siokra 1-4_Dt           AGCTCGATTCGGAGCTCGAACAAGACGAGTATAGCAGGAAACGACTATGATCCGATAGTT 600
T586_Dt                 AGCTCGATTCGGAGCTCGAACAAGACGAGTATAGCAGGAAACGACTATGATCCGATAGTT 600
Sipima 280_Dt           AGCTCGATTCGGAGCTCGAACAAGACGAGTATAGCAGGAAACGACTATGATCCGATAGTT 600
Pima A8_Dt              AGCTCGATTCGGAGCTCGAACAAGACGAGTATAGCAGGAAACGACTATGATCCGATAGTT 600
3-79_Dt                 AGCTCGATTCGGAGCTCGAACAAGACGAGTATAGCAGGAAACGACTATGATCCGATAGTT 600
Coker 315_At            AGCTCGATTCGGAGCTCGAACAAGCCGAGTAGAGCAGGAAACGACTATGATCCGATAGTT 597
MCU-5_At                AGCTCGATTCGGAGCTCGAACAAGCCGAGTAGAGCAGGAAACGACTATGATCCGATAGTT 597
Yumian1_At              AGCTCGATTCGGAGCTCGAACAAGCCGAGTAGAGCAGGAAACGACTATGATCCGATAGTT 597
Sicot 71_At             AGCTCGATTCGGAGCTCGAACAAGCCGAGTAGAGCAGGAAACGACTATGATCCGATAGTT 597
Siokra 1-4_At           AGCTCGATTCGGAGCTCGAACAAGCCGAGTAGAGCAGGAAACGACTATGATCCGATAGTT 597
T586_At                 AGCTCGATTCGGAGCTCGAACAAGCCGAGTAGAGCAGGAAACGACTATGATCCGATAGTT 597
Pima A8_At              AGCTCGATTCGGAGCTCGAACAAGCCGAGTAGAGCAGGAAACGACTATGATCCGATAGTT 597
3-79_At                 AGCTCGATTCGGAGCTCGAACAAGCCGAGTAGAGCAGGAAACGACTATGATCCGATAGTT 597
A-00507_A2              AGCTCGATTCGGAGCTCGAACAAGCCGAGTATAGCAGGAAACGACTATGATCCGATAGTT 597
YZ                      AGCTCGATTCGGAGCTCGAACAAGCCGAGTATAGCAGGAAACGACTATGATCCGATAGTT 597
M18                     AGCTCGATTCGGAGCTCGAACAAGCCGAGTATAGCAGGAAACGACTATGATCCGATAGTT 597
BM13H                   AGCTCGATTCGGAGCTCGAACAAGCCGAGTATAGCAGGAAACGACTATGATCCGATAGTT 597
                        ************************ ****** ***** ****** *** ***********

Gorai.002G244000_D5     GAATGTAACTATATTTTTCAATGAGGATGAGAATAACCCAGTTTCCACTCACTACTGGGAT 659
Coker 315_Dt            GAATGTAACTATATTTTTCAATGAGGATGAGAATAACCCAGTTTCCACTCACTACTGGGAT 651
MCU-5_Dt                GAATGTAACTATATTTTTCAATGAGGATGAGAATAACCCAGTTTCCACTCACTACTGGGAT 651
Yumian1_Dt              GAATGTAACTATATTTTTCAATGAGGATGAGAATAACCCAGTTTCCACTCACTACTGGGAT 651
Sicot 71_Dt             GAATGTAACTATATTTTTCAATGAGGATGAGAATAACCCAGTTTCCACTCACTACTGGGAT 651
TM-1_Dt                 GAATGTAACTATATTTTTCAATGAGGATGAGAATAACCCAGTTTCCACTCACTACTGGGAT 651
89004-64_Dt             GAATGTAACAATATTTTTCAATGAGGATGAGAATAACCCAGTTTCCACTCACTACTGGGAT 659
Siokra 1-4_Dt           GAATGTAACAATATTTTTCAATGAGGATGAGAATAACCCAGTTTCCACTCACTACTGGGAT 660
T586_Dt                 GAATGTAACAATATTTTTCAATGAGGATGAGAATAACCCAGTTTCCACTCACTACTGGGAT 660
Sipima 280_Dt           GAATGTAACAATATTTTTCAATGAGGATGAGAATAACCCAGTTTCCACTCACTACTGGGAT 660
Pima A8_Dt              GAATGTAACAATATTTTTCAATGAGGATGAGAATAACCCAGTTTCCACTCACTACTGGGAT 660
3-79_Dt                 GAATGTAACAATATTTTTCAATGAGGATGAGAATAACCCAGTTTCCACTCACTACTGGGAT 660
Coker 315_At            GAATGTAACAATATTTTTCAATGAGGATGAGAATAACCCAGTTTCCACTCACTACTGGGAT 657
MCU-5_At                GAATGTAACAATATTTTTCAATGAGGATGAGAATAACCCAGTTTCCACTCACTACTGGGAT 657
Yumian1_At              GAATGTAACAATATTTTTCAATGAGGATGAGAATAACCCAGTTTCCACTCACTACTGGGAT 657
Sicot 71_At             GAATGTAACAATATTTTTCAATGAGGATGAGAATAACCCAGTTTCCACTCACTACTGGGAT 657
Siokra 1-4_At           GAATGTAACAATATTTTTCAATGAGGATGAGAATAACCCAGTTTCCACTCACTACTGGGAT 657
T586_At                 GAATGTAACAATATTTTTCAATGAGGATGAGAATAACCCAGTTTCCACTCACTACTGGGAT 657
Pima A8_At              GAATGTAACAATATTTTTCAATGAGGATGAGAATAACCCAGTTTCCACTCACTACTGGGAT 657
3-79_At                 GAATGTAACAATATTTTTCAATGAGGATGAGAATAACCCAGTTTCCACTCACTACTGGGAT 657
A-00507_A2              GAATGTAACAATATTTTTCAATAAGGATGAGAATAACCCAGTTTCCACTCACTACTGGGAT 657
YZ                      GAATGTAACAATATTTTTCAATAAGGATGAGAATAACCCAGTTTCCACTCACTACTGGGAT 657
M18                     GAATGTAACAATATTTTTCAATAAGGATGAGAATAACCCAGTTTCCACTCACTACTGGGAT 657
BM13H                   GAATGTAACAATATTTTTCAATAAGGATGAGAATAACCCAGTTTCCACTCACTACTGGGAT 657
                        ********* ******** ** **************************************

Gorai.002G244000_D5     ATTCAACTCCCTTCTTATCCCTAA 683
Coker 315_Dt            ATTCAACTCCCTTCTTATCCCTAA 675
MCU-5_Dt                ATTCAACTCCCTTCTTATCCCTAA 675
Yumian1_Dt              ATTCAACTCCCTTCTTATCCCTAA 675
Sicot 71_Dt             ATTCAACTCCCTTCTTATCCCTAA 675
TM-1_Dt                 ATTCAACTCCCTTCTTATCCCTAA 675
89004-64_Dt             ATTCAACTCCCTTCTTATCCCTAA 683
Siokra 1-4_Dt           ATTCAACTCCCTTCTTATCCCTAA 684
T586_Dt                 ATTCAACTCCCTTCTTATCCCTAA 684
Sipima 280_Dt           ATTCAACTCCCTTCTTATCCCTAA 684
Pima A8_Dt              ATTCAACTCCCTTCTTATCCCTAA 684
3-79_Dt                 ATTCAACTCCCTTCTTATCCCTAA 684
Coker 315_At            ATTCAACTCCCTCCTTATCCCTAA 681
MCU-5_At                ATTCAACTCCCTCCTTATCCCTAA 681
Yumian1_At              ATTCAACTCCCTTCTTATCCCTAA 681
Sicot 71_At             ATTCAACTCCCTTCTTATCCCTAA 681
Siokra 1-4_At           ATTCAACTCCCTTCTTATCCCTAA 681
T586_At                 ATTCAACTCCCTCCTTATCCCTAA 681
Pima A8_At              ATTCAACTCCCTTCTTATCCCTAA 681
3-79_At                 ATTCAACTCCCTTCTTATCCCTAA 681
A-00507_A2              ATTCAACTCCCTTCTTATCCCTAA 681
YZ                      ATTCAACTCCCTTCTTATCCCTAA 681
M18                     ATTCAACTCCCTTCTTATCCCTAA 681
BM13H                   ATTCAACTCCCTTCTTATCCCTAA 681
                        ************ ***********
```

Fig. S6 Alignment of the protein sequences of GhOKRA from representative accessions used in this study. The amino acid change unique to the normal and okra leaf accessions is boxed (pink). The homeodomain and leucine-zippers are indicated by red and green bars, respectively. The Upland cotton accessions with okra or super-okra leaf are underlined.

```
Gorai.002G244000         MDWDGTIRPFISRLEPSLNFPSNYNYNQYPEGVEDMNNQGFEEAGNGLVPDLNMNGFMNN 60
Gh_OKRA_Dt_89004-64      MDWDGTIRPFISRLEPSLNFPSNYNYNQYPEGVEDMDNQGFEEAGNGLVPDLNMNGFMNN 60
Gh_OKRA_Dt_T586          MDWDGTIRPFISRLEPSLNFPSNYNYNQYPEGVEDMDNQGFEEAGNGLVPDLNMNGFMNN 60
Gh_OKRA_Dt_Siokra 1-4    MDWDGTIRPFISRLEPSLNFPSNYNYNQYPEGVEDMDNQGFEEAGNGLVPDLNMNGFMNN 60
Gh_OKRA_Dt_MCU-5         MDWDGTIRPFISRLEPSLNFPSNYNYNQYPEGVEDMNNQGFEEAGNGLVPDLNMNGFMNN 60
Gh_OKRA_Dt_Yumian1       MDWDGTIRPFISRLEPSLNFPSNYNYNQYPEGVEDMNNQGFEEAGNGLVPDLNMNGFMNN 60
Gb_OKRA_Dt_3-79          MDWDGTIRPFISRLEPSLNFPSNYNYNQYPEGVEDMDNQGFEEAGNGLVPDLNMNGFMNN 60
A_00507_A2               MDWNGTIRPFISRLEPSLNFPYNYNYNQYP-GVEDMNNQGFEEAGNGLVPDLNMNSFKNN 59
Gh_OKRA_At_T586          MDWDGTIRPFISRLEPSLNFPYNYNYNQYP-GVEDMNNQGFEEAGNGLVPDLNMNSFKNN 59
Gh_OKRA_At_Siokra 1-4    MDWNGTIRPFISRLEPSLNFPYNYNYNQYP-GVEDMNNQGFEEAGNGLVPDLNMNSFKNN 59
Gh_OKRA_At_MCU-5         MDWNGTIRPFISRLEPSLNFPYNYNYNQYP-GVEDMNNQGFEEAGNGLVPDLNMNSFKNN 59
Gh_OKRA_At_Yumian1       MDWDGTIRPFISRLETSLNFPYNYNYNQYP-GVEDMNNQGFEEAGNGLVPDLNMNSFKNN 59
                         ***:***********.***** ******** *****:******************.* **
```

HD domain

```
Gorai.002G244000         GKGNNNKKKRLTSDQLDSLERSFQEENKLDPDRKMKLSKELGLQPRQIAVWFQNRRARWK 120
Gh_OKRA_Dt_89004-64      GKGNNNKKKRLTSDQLDSLERSFQEENKLDPDRKMKLSKELGLQPRQIAVWFQNRRARWK 120
Gh_OKRA_Dt_T586          GKGNNNKKKRLTSDQLDSLERSFQEENKLDPDRKMKLSKELGLQPRQIAVWFQNRRARWK 120
Gh_OKRA_Dt_Siokra 1-4    GKGNNNKKKRLTSDQLDSLERSFQEENKLDPDRKMKLSKELGLQPRQIAVWFQNRRARWK 120
Gh_OKRA_Dt_MCU-5         GKGKNNKKKRLTSDQLDSLERSFQEENKLDPDRKMKLSKELGLQPRQIAVWFQNRRARWK 120
Gh_OKRA_Dt_Yumian1       GKGKNNKKKRLTSDQLDSLERSFQEENKLDPDRKMKLSKELGLQPRQIAVWFQNRRARWK 120
Gb_OKRA_Dt_3-79          GKGNNNKKKRLTSDQLDSLERSFQEENKLDPDRKMKLSKELGLQPRQIAVWFQNRRARWK 120
A_00507_A2               GKGNNKKKKRLTSDQLDSLERSFQEENKLDPDRKMKLSKELGLQPRQIAVWFQNRRARWK 119
Gh_OKRA_At_T586          GKGNNKKKKRLTSDQLDSLERSFQEENKLDPDRKMKLSKELGLQPRQIAVWFQNRRARWK 119
Gh_OKRA_At_Siokra 1-4    GKGNNKKKKRLTSDQLDSLERSFQEENKLDPDRKMKLSKELGLQPRQIAVWFQNRRARWK 119
Gh_OKRA_At_MCU-5         GKGNNKKKKRLTSDQLDSLERSFQEENKLDPDRKMKLSKELGLQPRQIAVWFQNRRARWK 119
Gh_OKRA_At_Yumian1       GKVNNKKKKRLTSDQLDSLERSFQEENKLDPDRKMKLSKELGLQPRQIAVWFQNRRARWK 119
                         ** :*:******************************************************
```

Leucine-zippers

```
Gorai.002G244000         AKQLEHSYNTLKHEYDIISIEKQKLQDEVMELKGMLVEQATRNQVSTVYKEISGGEIIES 180
Gh_OKRA_Dt_89004-64      AKQLEHSYNTLKHEYDIISIEKQKLQDEVMELKGMLVEQATRNQVSTVYKEISGGEIIES 180
Gh_OKRA_Dt_T586          AKQLEHSYNTLKHEYDIISIEKQKLQDEVMELKGMLVEQATRNQVSTVYKEISGGEIIES 180
Gh_OKRA_Dt_Siokra 1-4    AKQLEHSYNTLKHEYDIISIEKQKLQDEVMELKGMLVEQATRNQVSTVYKEISGGEIIES 180
Gh_OKRA_Dt_MCU-5         AKQLEHSYNTLKHEYDIISIEKQKLQDEVMELKGMLGDKEPS---------LHG---LQG 168
Gh_OKRA_Dt_Yumian1       AKQLEHSYNTLKHEYDIISIEKQKLQDEVMELKGMLGDKEPS---------LHG---LQG 168
Gb_OKRA_Dt_3-79          AKQLEHSYNTLKHEYDIISIEKQKLQDEVMELKGMLVEQATRNQVSTVYKEISGGEIIES 180
A_00507_A2               AKQLQHSYNTLKHEYDVIYMEKQMLQDEVMELKGMLMEQATRNQVSTVYKEISGGETIES 179
Gh_OKRA_At_T586          AKQLQHSYNTLKHEYDVTYMEKQMLQDEVMELKGMLMEQATRNQVSTVYKEISGGETIES 179
Gh_OKRA_At_Siokra 1-4    AKQLQHSYNTLKHEYDVTYMEKQMLQDEVMELKGMLMEQATRNQVSTVYKEISGGETIES 179
Gh_OKRA_At_MCU-5         AKQLQHSYNTLKHEYDVTYMEKQMLQDEVMELKGMLMEQATRNQVSTVYKEISGGETIES 179
Gh_OKRA_At_Yumian1       AKQLQHSYNTLKHEYDVTYMEKQMLQDEVMELKGMLMEQATRNQVSTVYKEISGGETIES 179
                         ****:***********:  :*** ************ :: .          : *   ::.

Gorai.002G244000         SSIRSSNKTSIAGNDYIR------------------------------------------ 198
Gh_OKRA_Dt_89004-64      SSIRSSNKTS--------IAETTMIR---------------------------------- 198
Gh_OKRA_Dt_T586          SSIRSSNKTSIAGNDYDPIVECNNIFNEDENNPVSTHYWDIQLPSYP 227
Gh_OKRA_Dt_Siokra 1-4    SSIRSSNKTSIAGNDYDPIVECNNIFNEDENNPVSTHYWDIQLPSYP 227
Gh_OKRA_Dt_MCU-5         NLRRRDHRK--------------------------------------- 177
Gh_OKRA_Dt_Yumian1       NLRRRDHRK--------------------------------------- 177
Gb_OKRA_Dt_3-79          SSIRSSNKTSIAGNDYDPIVECNNIFNEDENNPVSTHYWDIQLPSYP 227
A_00507_A2               SSIRSSNKPSIAGNDYDPIVECNNIFNKDENNPVSTHYWDIQLPSYP 226
Gh_OKRA_At_T586          SSIRSSNKPSRAGNDYDPIVECNNIFNEDENNPVSTHYWDIQLPPYP 226
Gh_OKRA_At_Siokra 1-4    SSIRSSNKPSRAGNDYDPIVECNNIFNEDENNPVSTHYWDIQLPSYP 226
Gh_OKRA_At_MCU-5         SSIRSSNKPSRAGNDYDPIVECNNIFNEDENNPVSTHYWDIQLPPYP 226
Gh_OKRA_At_Yumian1       SSIRSSNKPSRAGNDYDPIVECNNIFNEDENNPVSTHYWDIQLPSYP 226
                         .  * .::
```

Fig. S7 Phylogenetic analysis of all okra leaf accessions genotyped by the SNP chip. Twenty-eight (15 from the 85 used in confirmation of the okra leaf locus and 13 from the 92 used in KASP assay) okra leaf and six normal leaf *G. hirsutum* accessions were genotyped using the CottonSNP63K array and analysed using the SNPs flanking (6 Mbp) the okra leaf locus.

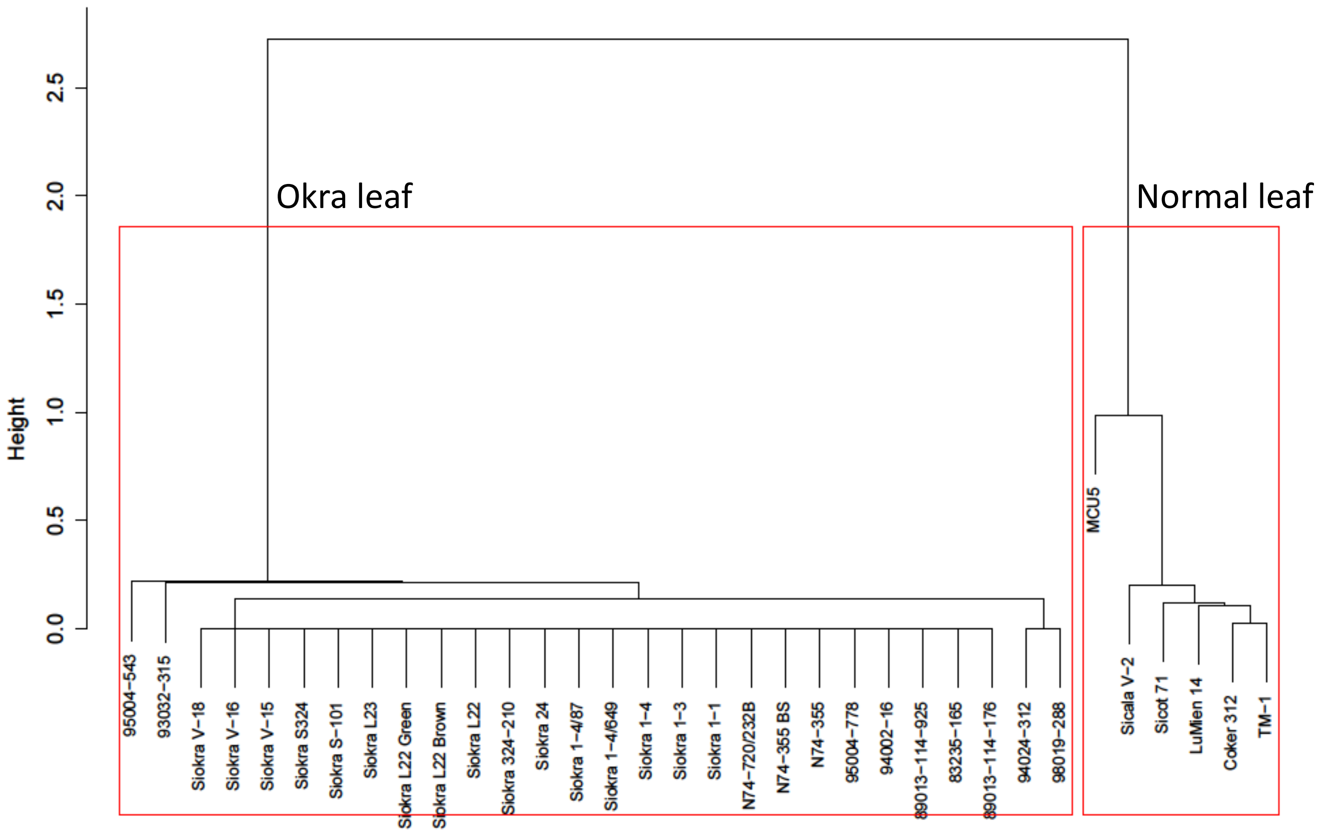

Fig. S8 Alignment of the promoter sequences of *GhOKRA-Dt* from MCU-5 and Siokra 1-4. The translation start codon in underlined in bold type.

```
Gorai.002G244000_P            GGTAACACACATCGCAATCCCTCGGGTTCGAACAT-AAAACCGTATAACG 49
GhOKRA_P_MCU-5_Dt             GGTAACACACATCGCAATCCCTCGGGTTCGAACATTAAAACCGTATAACG 50
GhOKRA_P_Siokra 1-4_Dt        GGTAACACACATCGCAATCCCTCGGGTTCGAACATTAAAACCGTATAACG 50
                              *********************************** **************

Gorai.002G244000_P            GATATAAAAGATACATTAGTGAAGGCTGGTAATTATATGTGGTTGTTTTC 99
GhOKRA_P_MCU-5_Dt             GATATAAAAGATACATTAGTGAAGGCTGGTAATTATATGTGGTTGTTTTC 100
GhOKRA_P_Siokra 1-4_Dt        GATATAAAAGATACATTAGTGAAGGCTGGTAATTATATGTGGTTGTTTTC 100
                              **************************************************

Gorai.002G244000_P            TGTTTGTATTTTGCACGGTGATGGTTCGTCCTGTTGTGTGTATTGCCCTA 149
GhOKRA_P_MCU-5_Dt             TGTTTGTATTTTGCACGGTGATGGTTCGTCCTGTTGTGTGTATTGCCCTA 150
GhOKRA_P_Siokra 1-4_Dt        TGTTTGTATTTTGCACGGTGATGGTTCGTCCTGTTGTGTGTATTGCCCTA 150
                              **************************************************

Gorai.002G244000_P            ATATATTGCTTGGTTGTATGGTCGTTGGTTTTGTTGGGGTTGCAGCCTTT 199
GhOKRA_P_MCU-5_Dt             ATATATTGCTTGGTTGTATGGTCGTTGGTTTTGTTGGGGTTGCAGCCTTT 200
GhOKRA_P_Siokra 1-4_Dt        ATATATTGCTTGGTTGTATGGTCGTTGGTTTTGTTGGGGTTGCAGCCTTT 200
                              **************************************************

Gorai.002G244000_P            GCAGTACTGGGTCTAGTATGGTGCTTTCTCCGGGTGCTATCTTTCAGTAC 249
GhOKRA_P_MCU-5_Dt             GCAGTACTGGGTCTAGTATGGTGCTTTCTCCGGGTGCTATCTTTCAGTAC 250
GhOKRA_P_Siokra 1-4_Dt        GCAGTACTGGGTCTAGTATGGTGCTTTCTCCGGATGCTATCTTTCAGTAC 250
                              ********************************* ****************

Gorai.002G244000_P            TAATTTTTGGTTGTTGGGATCAACCTTTTTATTGTCGTAAAAGAT----- 294
GhOKRA_P_MCU-5_Dt             TAATTTTTGGTTGTTGGGATCAACCTTTTTATTGTCGTAAAAGATCATGC 300
GhOKRA_P_Siokra 1-4_Dt        TAATTTATGGTTGTTGGGATCAACCTTTTTATTGTCGTAAAAGATCATGC 300
                              ****** **************************************

Gorai.002G244000_P            --------------------------------------------------
GhOKRA_P_MCU-5_Dt             ATGTCATGTCTGAGAAATAAACAAAAAC---------------------- 328
GhOKRA_P_Siokra 1-4_Dt        ATGTCATGTCTGAGAAATAAACAAAAACAAGGACAAAATAATGAACGGTT 350


Gorai.002G244000_P            --------------------------------------------------
GhOKRA_P_MCU-5_Dt             --------------------------------------------------
GhOKRA_P_Siokra 1-4_Dt        GAAAAAGTGAGTCATGCAAGGACGAAGATTCAGTAATAAAATGAAAGGCA 400


Gorai.002G244000_P            --------------------------------------------------
GhOKRA_P_MCU-5_Dt             --------------------------------------------------
GhOKRA_P_Siokra 1-4_Dt        TATATTATATTGTCAGCGATTAAAAGATCATGCATGTCATGTCTGAGAAA 450


Gorai.002G244000_P            -----------AAGGACAAAATAATGAACGGTTGAAAAAGTGAGTCATGC 333
GhOKRA_P_MCU-5_Dt             -----------AAGGACAAAATAATGAACGGTTGAAAAAGTGAGTCATGC 367
GhOKRA_P_Siokra 1-4_Dt        TAAACAAAAACAAGGACAAAATAATGAACGGTTGAAAAAGTGAGTCATGC 500
                                         ***************************************

Gorai.002G244000_P            AAGGACGAAGATTCAGTAAAAAAATGAAAGGCATATATTATATCGTCAGC 383
GhOKRA_P_MCU-5_Dt             AAGGACGAAGATTCAGTAATAAAATGAAAGGCATAGATTATATTGTCAGC 417
GhOKRA_P_Siokra 1-4_Dt        AAGGACGAAGATTCAGTAATAAAATGAAAGGCATATATTATATTGTCAGC 550
                              ******************* *************** ******* ******

Gorai.002G244000_P            GATTAGGTTTTTTAGATAGAGACCCATGATTGTTCATTTCCAATTCTCTT 433
GhOKRA_P_MCU-5_Dt             GATTAGGTTTATTAGATAGAGACCCATGATTGTTCATTTCCAATTCTCTT 467
GhOKRA_P_Siokra 1-4_Dt        GATTAGGTTTATTAGATAGAGACCCATGATTGTTCATTTCCAATTCTCTT 600
                              ********** ***************************************

Gorai.002G244000_P            TTAACATCACAAAATGAGTTGTTAAGTGGTTTTTTTTTAAATGTTATATT 483
GhOKRA_P_MCU-5_Dt             TTAACATCACAAAATGAGTTGTTAAGTGGTTTTTTTTTAAATGTTATATT 517
GhOKRA_P_Siokra 1-4_Dt        TTAACATCACAAAATGAGTTGTTAAGTGGTTTTTTTTTAAATGTTATATT 650
                              **************************************************

Gorai.002G244000_P            AGCGAAATTTAAGTGATGATTTGACGATTGATGTAATGAATGAGTA---- 529
GhOKRA_P_MCU-5_Dt             AGCGAAGTTTAAGTGATGATTTGACGATTGATGTAATGAATGAGTATTTA 567
GhOKRA_P_Siokra 1-4_Dt        AGCGAAGTTTAAGTGATGATTTGACGATTGATGTAATGAATGAGTATTTA 700
                              ****** ***************************************
```

```
Gorai.002G244000_P          ----------GAAGAAGATATTTTAAATCCAAGTTGATTTAACGATCAAT 569
GhOKRA_P_MCU-5_Dt           TCAATGAGTAAAAGAAAATATTTTAAATTCAAGTTGATTTAACGATCAAT 617
GhOKRA_P_Siokra 1-4_Dt      TCAATGAGTAAAAGAAAATATTTTAAATTCAAGTTGATTTAACGATCAAT 750
                                       ***** *********** *********************

Gorai.002G244000_P          ATCAAATATTGACAAATAAAGCTATTTGAATTTTGGTTAACTGATACTTG 619
GhOKRA_P_MCU-5_Dt           ATCAGATATTGACAAATAAAACTATTTGAATTTTGGTTAACTGATACTTG 667
GhOKRA_P_Siokra 1-4_Dt      ATCAGATATTGACAAATAAAACTATTTGAATTTTGGTTCACTGATACTTG 800
                            **** *************** ***************** ***********

Gorai.002G244000_P          ATGTTCAAAGATATTTTCTAAAAAAAAATTGAACTGTAAAAAGGAAATTG 669
GhOKRA_P_MCU-5_Dt           ATGTTCAAAGATATTTCCTGAAAAAAAATTGAACTGTAAAAAGGAAATTG 717
GhOKRA_P_Siokra 1-4_Dt      ATGTTCAAAGATATTTCCTGAAAAAAAATTGAACTGTAAAAAGGAAATTG 850
                            **************** ** ******************************

Gorai.002G244000_P          AAGAAAAACTTTTGTTTGATGAGGGATTGTAAATGTACAAGATCATATGG 719
GhOKRA_P_MCU-5_Dt           AAGAAAAACTTTTGTTTGATGAGGGATTGTAAATGTACAAGATCATATGG 767
GhOKRA_P_Siokra 1-4_Dt      AAGAAAAACTTTTGTTTGATGAGGGATTGTAAATGTACAAGATCATATGG 900
                            **************************************************

Gorai.002G244000_P          TAATTTATCTCTTTTTCTAATTAAAAAAAGTTAAAAGTAAGCCTAAAACC 769
GhOKRA_P_MCU-5_Dt           TAATTTATCTCTTTTTCTAATTAAAAAAAGTTAAAAGTAAGCCTAAAACC 817
GhOKRA_P_Siokra 1-4_Dt      TAATTTATCTCTTTTTCTAATTAAAAAAAGTTAAAAGTAAGCCTAAAACC 950
                            **************************************************

Gorai.002G244000_P          TAATCAACTAAACCAATTAAACCAACCTATTGTCATGGTTCTTCTAATTA 819
GhOKRA_P_MCU-5_Dt           TAATCAACTAAACCAATTAAACCAGCCTATTGTCATGGTTCTTCTAATTA 867
GhOKRA_P_Siokra 1-4_Dt      TAATCAACTAAACCAATTAAACCAACCTATTGTCATGGTTCTTCTAATTA 1000
                            ************************ *************************

Gorai.002G244000_P          ACAGACAAGTCCACCCTCTCTCCATCTCCCTTTAAAATCACACGTTAAGG 869
GhOKRA_P_MCU-5_Dt           ACAGACAAGTCCACCCTCTCTCCATCTCCCTTTAAAATCACACGTTAAGG 917
GhOKRA_P_Siokra 1-4_Dt      ACAGACAAGTCCACCCTCTCTCCATCTCCCTTGAAAATCACACGTTAAGG 1050
                            ******************************** *****************

Gorai.002G244000_P          CATCGTTGCATGCTCCCCCCAACAGTCAACCCCTTAACAACAGTCAACCC 919
GhOKRA_P_MCU-5_Dt           CATTGTTGCATGCTCCCCCCAACAGTCAACCCCTTCACAACAGTCAACCC 967
GhOKRA_P_Siokra 1-4_Dt      CATTGTTGTATGCTCCCCCCAACAGTCAACCCCTTCACAACAGTCAACCC 1100
                            *** **** ************************** **************

Gorai.002G244000_P          CCCTTTCAGTTCTGGTCTGCCCCCCATTGCTATGCTACTTTAGGCTTACT 969
GhOKRA_P_MCU-5_Dt           CCCTTTCAGTTCTGGTCTGCTCCCCATTGCTATGCTACTTTAGGCTCACT 1017
GhOKRA_P_Siokra 1-4_Dt      CCCTTTCAGTTCTGGTCTGCTCCCCATTGCTATGCTACTTTAGGCTCACT 1150
                            ******************** ************************* ***

Gorai.002G244000_P          CTTCGGGCAGCTTCGTTCAGTTCTGGTGGTTCCATTTATATATACAGATA 1019
GhOKRA_P_MCU-5_Dt           CTTTGGGCAGCTTCGTTCAGTTCTGGTGGTTCCATTTATATATACAGATA 1067
GhOKRA_P_Siokra 1-4_Dt      CTTTGGGCAGCTTCGTTCAGTTCTGGTGGTTCCATTTATATATACAGATA 1200
                            *** **********************************************

Gorai.002G244000_P          TACTCGCTTTCTTCATTCAAAGAAATGGATTGGGATGGCACCATTCGACC 1069
GhOKRA_P_MCU-5_Dt           TACTCGCTTTCTTCATTCAAAGAAATGGATTGGGATGGCACCATTCGACC 1117
GhOKRA_P_Siokra 1-4_Dt      TACTCGCTTTCTTCATTCAAAGAAATGGATTGGGATGGCACCATTCGACC 1250
                            **************************************************

Gorai.002G244000_P          CTTTATTTCACGAC 1083
GhOKRA_P_MCU-5_Dt           CTTTATTTCACGAC 1131
GhOKRA_P_Siokra 1-4_Dt      CTTTATTTCACGAC 1264
                            **************
```
